# Molecular Imaging of Hydrolytic Enzymes Using PET and SPECT

**DOI:** 10.1177/1536012117717852

**Published:** 2017-09-20

**Authors:** Brian P. Rempel, Eric W. Price, Christopher P. Phenix

**Affiliations:** 1 Department of Science, Augustana Faculty, University of Alberta, Edmonton, Alberta, Canada; 2 Department of Chemistry, University of Saskatchewan, Saskatoon, Saskatchewan, Canada; 3 Biomarker Discovery, Thunder Bay Regional Health Research Institute, Thunder Bay, Ontario, Canada

**Keywords:** positron emission tomography, PET, single-photon emission computer tomography, SPECT, esterase, lipase, sulfatase, glycosidase, histone deacetylase, fatty acid amide hydrolase

## Abstract

Hydrolytic enzymes are a large class of biological catalysts that play a vital role in a plethora of critical biochemical processes required to maintain human health. However, the expression and/or activity of these important enzymes can change in many different diseases and therefore represent exciting targets for the development of positron emission tomography (PET) and single-photon emission computed tomography (SPECT) radiotracers. This review focuses on recently reported radiolabeled substrates, reversible inhibitors, and irreversible inhibitors investigated as PET and SPECT tracers for imaging hydrolytic enzymes. By learning from the most successful examples of tracer development for hydrolytic enzymes, it appears that an early focus on careful enzyme kinetics and cell-based studies are key factors for identifying potentially useful new molecular imaging agents.

## PET and SPECT Imaging

Positron emission tomography (PET) and single-photon emission computed tomography (SPECT) are nuclear-based molecular imaging modalities. They are used for noninvasively tracking an injected molecule labeled with a radioactive isotope to study its spatial and temporal distribution in a living subject to gain fundamental information about disease biology.^1^ Today, PET-CT and SPECT-CT instruments are both available as dual imaging systems enabling anatomical computed tomography (3-D X-ray) images to be overlaid with the radioactive signal. Such techniques enable real-time imaging of physiological processes such as blood flow and cardiac function, but they truly excel at “molecular imaging” of unique disease-specific metabolic processes including the activation of enzymatic pathways and cellular processes such as receptor expression, angiogenesis, and apoptosis. Positron emission tomography and SPECT are used extensively in both preclinical and clinical settings due to their high sensitivity and total body penetrance, which enable detection of the tracer in any tissues or organs in the full body. With careful design and selection of the radiotracer, these scans can serve as the equivalent of a noninvasive whole-body biopsy for a given process, as the nature of nuclear imaging scans allows quantitative measurement of radiotracer distribution. Owing to the high sensitivity of PET and SPECT, only microdoses of radiotracer are needed (final biological concentrations typically nM-pM), which generally avoids unwanted pharmacological side effects of the chemical probe. However, the design of PET and SPECT tracers capable of imaging enzymatic targets is an enormous challenge due to the need for agents that have high affinity and specificity toward the target enzyme, possess good clearance properties to reduce background signal from nonspecific tissue uptake, and are metabolically stable. In addition, the preparation, purification, and quality control of radiotracers are demanding due largely to the short half-life of many commonly used isotopes (especially for PET). Other challenges include poor penetration of the tracer into specific tissues such as tumors or organs such as the brain, limitations associated with using animal models as a surrogate to human diseases, and the difficulty and costs of obtaining sufficient data for Food and Drug Administration approval.^2^ The versatile functionality and general success of nuclear imaging and new radiopharmaceuticals have led to increasing availability of PET and SPECT cameras at many research centers and urban hospitals, further increasing the need for new and innovative molecular imaging agents.

Positron emission tomography utilizes isotopes with an energetically unstable proton to neutron ratio (eg, ^11^C, ^18^F, ^68^Ga, ^64^Cu, ^89^Zr), which are often produced in a cyclotron or a generator system, where the generator parent isotope may be produced via cyclotron or nuclear reactor. These radioisotopes decay through emission of a positron (the antimatter equivalent of an electron bearing a positive charge) that travels a short distance before colliding with an electron. Mutual annihilation of both particles results in the release of 2 coincident 511 keV gamma rays, which are detected simultaneously by a PET camera (coincidence detectors).

For PET imaging, ^18^F has traditionally been the isotope of choice because of its 109.7-minute half-life, ready availability at virtually every medical cyclotron facility in the world, and good image resolution as a result of low positron energy. Compared to ^11^C with its 20-minute half-life, the longer lived ^18^F isotope allows for the preparation of a wider range of radiotracers (with well-established radiolabeling chemistry) and provides more working time for purification, quality control, and distribution to nearby hospital radiopharmacies while still possessing a short enough half-life to minimize radiation dose to patients. Another attractive property of ^18^F compared to the radiometals is that it can be seamlessly substituted into small organic molecules as the change from a C-H or C-OH to a C-F bond is relatively conservative in terms of bond length, bond strength, and atomic radius. Although ^18^F is a near-perfect match for labeling small molecules intended for imaging, the use of radiometals has seen a surge of interest with larger drug vectors such as peptides, antibodies, and nanoparticles.^3^

As a consequence of their short half-lives, PET isotopes such as ^11^C and ^18^F require production at a nearby cyclotron facility, which significantly increases the costs associated with PET imaging. Long half-life isotopes for PET (eg, ^89^Zr, ^124^I) and SPECT (eg, ^111^In, ^123^I, ^131^I) are available but are not always as structurally compatible with small molecule drugs as either ^11^C or ^18^F due to their large size or need for chelation (eg, ^18^F is bioisosteric with hydrogen, bulky chelators are not).

Compared to SPECT, PET has higher sensitivity and therefore requires preparation and injection of a lower dose of radiotracer, is quantitative for more accurate pretherapy scouting scans and dosimetry calculations, and has fewer image artifacts leading to better quality images. Positron emission tomography is generally considered a superior technique for molecular imaging. In contrast, SPECT isotopes are longer lived (eg, ^99m^Tc *t*_1/2_ = 6.00 hours; ^123^I *t*_1/2_ = 13.2 hours, ^111^In *t*_1/2_ = 2.8 days) and decay through the direct emission of single gamma ray photons that are detected directly with pinhole cameras through collimators, which greatly reduces sensitivity when compared to noncollimated coincidence detection of PET. With SPECT cameras located in virtually every nuclear medicine department worldwide, SPECT represents ∼90% of all nuclide-based diagnostic medicine despite having inferior image quality and lower sensitivity compared to PET. Among the available SPECT isotopes, ^99m^Tc is the most commonly used (∼75% of all nuclear diagnostic tests) due to the low cost of reactor-produced ^99^Mo and the small and easily distributed ^99^Mo/^99m^Tc generator system. These generators are purchased by hospitals and research centers where the parent isotope ^99^Mo remains trapped at the top of the generators’ stationary phase, continually decaying into a supply of ^99m^Tc. The ^99m^Tc can be eluted (referred to as “milking”) daily for about a week and used directly with radiopharmaceutical kits for rapid production of the SPECT radiotracer. A majority of the world’s ^99^Mo supply is produced from highly enriched uranium in a handful of nuclear reactors: the National Research Universal reactor in Canada, High Flux Reactor in the Netherlands, Osiris in France, Belgium Reactor-2 (BR-2) in Belgium, and South Africa Fundamental Atomic Research Installation (SAFARI-1) in South Africa. As well, low-enrichment uranium is being used to produce ^99^Mo at a small number of reactors, including Australia’s Open Pool Australian Lightwater (OPAL) reactor.^4,5^ Despite its wide availability, ^99m^Tc requires most radiotracers to incorporate a bulky chelating group and is therefore not commonly used to label small molecules intended to image enzyme activity.

## Imaging Hydrolytic Enzymes

Hydrolases (abbreviated Enzyme Commission number (EC) 3 in the International Union of Biochemistry and Molecular Biology Enzyme Classification System) are a large family of enzymes responsible for breaking down biological molecules through addition of water followed by fragmentation of the substrate.^6^ Hydrolytic enzymes have a variety of critical roles in normal cell and tissue function but are also important in a large number of disease processes, including viral, parasitic, or bacterial infection, cancer, neurodegenerative diseases, and addiction. Tremendous efforts have been made in developing radiotracers to image in vivo hydrolase activity in animals and humans, with the goals of identifying enzyme-based biomarkers for diagnosing disease, understanding the role that a specific enzyme may play in disease-promoting processes, or identifying aberrant enzyme activity as a putative drug target. Indeed, many PET/SPECT agents have been developed to image esterases (EC 3.1), glycosylases (EC 3.2), proteases (EC 3.4), other carbon–nitrogen cleaving enzymes (EC 3.5), and, to our knowledge in only 1 case, ether hydrolases (EC 3.3). This review is focused on describing examples and highlighting the various strategies recently published in the scientific literature to image hydrolase enzyme activity using molecular imaging. In the case of hydrolytic enzymes recently reviewed in a comprehensive manner, an interested reader is directed to relevant articles without further comment. Furthermore, enzymes with hydrolytic activity that is secondary to the primary function of the protein, for example, an enzyme that couples adenosine diphosphate hydrolysis to another reaction, have been excluded.

## General Considerations for the Design of Enzyme Imaging Agents

The design and development of PET/SPECT tracers that can image enzyme activity can be classified into 3 broad categories, described below.

### Substrates

A key design feature of a substrate-based radiotracer for a hydrolase is that the enzyme activation of the radiolabeled substrate creates 2 fragments. One of the product fragments produced must both retain the radioactive label (eg, ^18^F) and become trapped in nearby cells or tissues so that the accumulation of the radioactive signal correlates with areas of high enzymatic processing (and high enzymatic activity). It is important to note that radioactive PET/SPECT images do not provide any information about the chemical state of the isotopes (eg, bound to tracer or not), and for example, isotope that is “lost” from the parent radioactive drug (eg, ^18^F being released, or on the wrong side of a hydrolyzed substrate) is indistinguishable from tracer-bound isotope. One of the major challenges in design of a substrate-based imaging agent is selectivity for the target enzyme. Designing a substrate-based tracer that will only reflect the activity of a single enzyme is challenging since closely related enzymes (isozymes) often recognize similar substrates and have therefore evolved very similar active site structures comprised of conserved amino acid residues. As well, a substrate must be processed with very high efficiency so that even at tracer levels of the substrate, sufficient probe is hydrolyzed and enough radioactive products accumulate to enable detection of enzyme activity prior to elimination or extensive decay of the radioisotope (eg, 1-4 hours postinjection for ^18^F). A major hurdle to overcome in the substrate-based approach is in the selection of the radiolabeled fragment that must undergo cellular or metabolic trapping: It must be easily radiolabeled, compatible with the enzyme active site, and reliably accumulate at the site of enzyme activity. Common strategies developed for trapping radiolabeled reporter groups include precipitation of hydrophobic products, intracellular accumulation of charged products, and reaction to form a covalent bond with a cellular component.^7-10^ Although not discussed here, substrate-based probes designed to image kinases have seen tremendous success because the phosphorylated product becomes ionically trapped inside cells (an ideal example of this approach is the phosphorylation of [^18^F]2-fluoro-2-deoxyglucose ([^18^F]FDG) by hexokinase).^11,12^ Despite these challenges, substrate-based radiotracers have the very important advantage of signal amplification. An individual enzyme molecule can turn over many probe molecules, leading to a high accumulation of radioactive signal in the region of enzyme activity. As well, in many disease processes, aberrant enzyme catalytic activity is observed and is critical for progression. Given that human enzyme activity is tightly controlled through complex posttranslational modifications and not expression levels alone, substrate-based radiotracers can be used with noninvasive PET imaging to reveal only those enzymes that are catalytically active.

### Reversible Inhibitors

This class of enzyme imaging tracer relies on the development of a potent radioactive ligand that noncovalently binds to a target with very high affinity (generally nM *K_i_*). Since the radioactive ligand will bind to the desired enzyme in a reversible manner, the PET/SPECT tracer will accumulate in areas with high levels of target enzyme leading to a strong radioactive signal. In tissues with low enzyme concentration, the tracer can diffuse away and is eliminated from the body (eg, urine/renal clearance), effectively reducing background uptake and improving image contrast. Frequently, inhibitors designed as therapeutics can serve as the inspiration for the design of reversible inhibitor-based radiotracers. Indeed, some failed drug candidates suffering from undesirable side effects or rapid metabolism can be converted into excellent tracers once radiolabeled analogues can be prepared. As radiopharmaceuticals are administered in tiny quantities as “microdoses,” pharmacological toxicity is far less of a concern. Another advantage of this approach is that potent ligands can be designed to bind at “allosteric sites” on the target enzyme that have often evolved as a specific way of controlling metabolic rates through inhibiting key enzymes. Given that allosteric sites often bind natural ligands specific to a metabolic pathway, tracers that occupy a unique allosteric site may be highly selective to the target enzyme over closely related homologous enzymes.^13^ Reversible ligands image enzyme expression rather than enzyme activity since the ligand binds in a 1:1 ratio with enzyme regardless of whether the enzyme is catalytically active in the local environment.^14,15^

### Irreversible Inhibitors

The third class of tracer molecule is irreversible inhibitors. These compounds are substrate analogues that, once activated by the target enzyme’s catalytic machinery, generate a reactive intermediate that covalently attaches to a nucleophilic amino acid residue typically in the enzyme’s active site. Enzyme activity is permanently inhibited either because a catalytic residue no longer functions as needed or because the inhibitor physically blocks the active site. Because these inhibitors rely on catalytic activity but also irreversibly tag the target enzyme, they image enzyme activity (as opposed to expression levels) with a 1:1 inhibitor to enzyme ratio. Since a stable enzyme–tracer complex forms, the radioactive signal is stably attached to the enzyme and therefore cannot diffuse away from area of enzyme activity. One feature of imaging agents based on irreversible inhibitors that is fundamentally different from substrate-based tracers is that a large number of substrate molecules can be processed by each individual enzyme which results in signal amplification over time, whereas irreversible inhibitors are limited to a 1:1 tracer to enzyme ratio.

A drawback of this approach is that the inhibitor efficiency must be carefully tuned since compounds that are too potent will instantaneously react with the target enzyme producing images that can reflect blood flow instead of areas of high enzyme activity. The behavior of potent irreversible inhibitors to image blood flow results from the rapid labeling of the target enzyme in highly vascularized tissues that occurs much faster than the rate of diffusion through tissues that have low enzyme expression. In this scenario, the limiting reagent is the radiotracer—meaning the inhibitor reacts with only a small proportion of the available enzyme molecules and imaging experiments are therefore unable to distinguish between areas of high or low enzyme activity.^14,15^

Two enzymes whose activity and expression have been successfully imaged with all 3 approaches are monoamine oxidases A and B (MAO A/B). These flavin-dependent oxidoreductase (EC 1.4.3.4) enzymes have been very well studied by Fowler and coworkers, and a number of imaging agents that are (1) substrates, (2) reversible, and (3) irreversible inhibitors have been described.^15^

It is also possible to image an enzyme using a radiolabeled monoclonal antibody. The benefits and challenges surrounding PET/SPECT imaging using antibodies are different from those encountered in small molecule development, including the higher potential specificity, longer times needed to accumulate specific signal in target tissues (imaging is usually feasible after 24-74 hours), possibility for development of an immune response to the imaging agent (eg, murine vs human antibodies), and inability for antibodies to natively cross cell membranes or cross the blood–brain barrier (BBB). Since the fundamental design of antibody-based radiotracers is different from imaging with small molecules,^16-19^ this review will focus only on small molecule radiotracers. For a more complete description of various design strategies for PET imaging probes, the interested reader should consult specific reviews on that topic.^8-10^

## Enzyme Kinetics for Tracers

The measurable parameters in an in vitro enzyme kinetic experiment are also different for each of the 3 classes of imaging agent. Each type of imaging agent has unique kinetic properties that can be measured using experiments conducted in vitro with recombinant enzymes or cultured cells. For a good introduction to the basics of enzyme kinetics, see the study by Cook and Cleland.^20^

### Substrates

**Figure 1.**
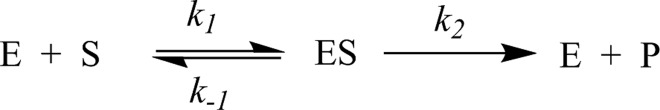
Kinetic scheme for Michaelis-Menten kinetics, in which E represents free enzyme, S represents free substrate, ES represents the enzyme–substrate complex, and P represents product.

Substrates for hydrolytic enzymes can be treated using classic Michaelis-Menten kinetics.^21^ The Michaelis-Menten equation describes a kinetic scheme in which a single substrate rapidly binds to an enzyme and is transformed into product in a single, irreversible step. A kinetic scheme representing this process is shown in Figure 1.

The equation that describes this process for velocity of the reaction (*v*) at concentration *S* is:

1 $$v=\frac{v_{\text{max}}\left[S\right]}{K_{m}+\left[S\right]}.$$

In this equation, the important parameters are *V*_max_ and *K_m_*. *V*_max_ is the maximal rate of reaction and is dependent on enzyme concentration. Dividing *V*_max_ by the total enzyme concentration yields the important parameter *k_cat_*, which represents the rate of the first committed chemical step of the reaction at saturating substrate concentrations. *K_m_* is the concentration of substrate needed to reach half the maximum rate (*V*_max_) and represents the affinity of the substrate for the Michaelis complex (ES). A low *K_m_* value indicates that the enzyme has a high affinity for the substrate, meaning that even at low concentrations of a radiotracer, appreciable levels of hydrolysis will be occurring. In contrast, a high *K_m_* value indicates the enzyme requires a high concentration of radiotracer to reach high levels of substrate turnover. *K_m_*, the Michaelis constant, is sometimes mistakenly conflated with the dissociation constant (*K_d_* value). These values are not equivalent, since *K_d_* = *k*_−1_/*k*_1_, while *K_m_* = (*k*_2_ + *k*_−1_/*k*_1_), therefore as *k_cat_* (which is = *k*_2_ in Michaelis-Menten kinetics) increases, the value of *K_m_* will progressively get larger than the *K_d_* value. In the limiting case where *k_cat_* for a substrate approaches 0, then the value of *K_m_* approaches *K_d_* and the small molecule is behaving as a potential inhibitor for the enzyme (discussed below). The ratio of *k_cat_*/*K_m_* is the enzymatic efficiency, representing the most valuable measurement for evaluating substrates intended as tracers for imaging enzyme activity. This ratio is a second-order rate constant for reaction of free enzyme and free substrate to form product and accounts for both the binding affinity of the substrate and the catalytic efficiency of the enzyme for the substrate.^22^ A high ratio for *k_cat_*/*K_m_* indicates that a substrate will be processed efficiently by the enzyme even at physiological concentrations and tracer levels of the radioactive substrate, which is a critical parameter for successful radiotracers (Table 1).

**Table 1.** Key Kinetic Values for the Discussion of Enzyme-Targeted Molecular Imaging Agents.

Kinetic Value	Type	Definition
IC_50_	Binding measure	Concentration of substrate at which enzyme activity is reduced by 50% under given assay conditions
*V*_max_	Maximum rate	Maximum rate of enzyme reaction
*K_i_*_,_ reversible	Inhibition constant	Equilibrium constant for binding of the inhibitor, ratio of the rate constant for enzyme–inhibitor dissociation divided by the ratio of enzyme–inhibitor association (*k_off_*/*k_on_*)
*K_i_*_,_ irreversible	Inhibition constant	Concentration at which the inactivation reaction proceeds at 50% of the maximum velocity (analogous to *K_m_* for a substrate)
*k_i_*	Rate	Rate constant for the reaction that generates the inactivated form of the enzyme (EI*) once the enzyme is saturated by the irreversible inhibitor (analogous to *k_cat_* for a substrate)
*K_m_*	Michaelis constant	Concentration of substrate needed to reach half the maximum rate (*V*_max_)
*k_cat_*	Rate	Rate of the first committed chemical step of the reaction at saturating substrate concentrations
*k_on_*	Rate	Rate of substrate binding to enzyme active site
*k_off_*	Rate	Rate of substrate dissociating from enzyme active site

In practice, potential substrates designed as tracers for imaging enzyme activity should have *k_cat_* and *K_m_* values rather than % activity measured, since *k_cat_* and *K_m_* values are independent of enzyme and substrate concentration. Measurements of % activity may be a useful guide for evaluating a series of substrates to select the most efficient, but without a proper kinetic characterization, it will be impossible to tell whether increases in efficiency arise from faster processing of the substrate (increased *k_cat_*) or improved binding (roughly reflected by *K_m_*). Furthermore, reporting % activity makes it very difficult to directly compare substrate efficiencies for assays performed in different laboratories, and so standardized *k_cat_* and *K_m_* values are far more useful.

The ideal substrates used for PET/SPECT imaging should have high *k_cat_* values, indicating the enzyme efficiently processes the substrate, and low *K_m_* values, indicating the enzyme requires a low concentration of substrate for efficiently processing. It can be assumed that at tracer levels (nM-pM), the [Substrate]<<*K_m_*, meaning that measuring *k_cat_* or *K_m_* alone will not provide information on overall efficiency. The *k_cat_*/*K_m_* value is of the most value for evaluating substrates as potential imaging agents, since this second-order rate constant describes the rate of free enzyme and free substrate converting to product at low substrate concentrations and takes into account both substrate binding and turnover rates.^22^ The challenge here is that many potential substrates cannot be easily assayed unless they happen to incorporate a reporter group, like a chromophore or fluorophore. In addition, sufficient levels of nonradioactive probe as well as access to recombinant enzyme or the development of a cell-based kinetic assay are needed for full kinetic characterization and represent a significant hurdle in many cases.

### Reversible Inhibitors

**Figure 2.**
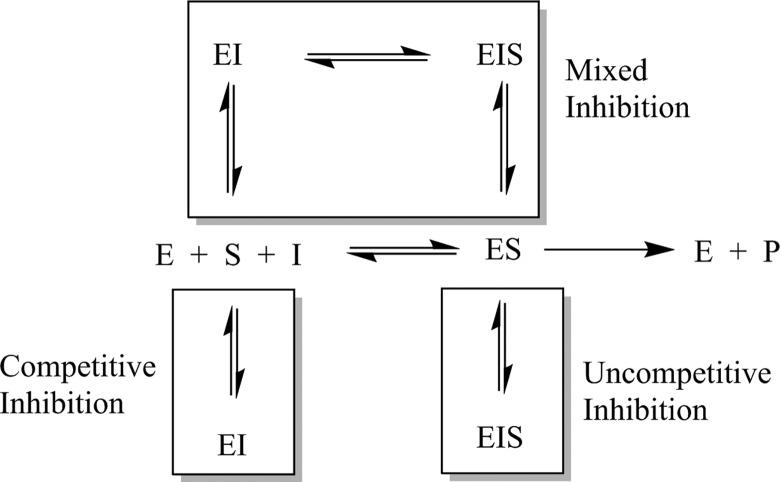
Kinetic scheme depicting enzyme-catalyzed turnover of substrate (center row) and various modes of reversible inhibition.

Reversible inhibitors bind to the enzyme and interfere with catalytic turnover of substrate. Reversible inhibitors can be classified as competitive (increases the apparent *K_m_* for a substrate, indicating it requires higher substrate levels), uncompetitive (decreases the apparent *k_cat_* for a substrate, indicating a decrease in efficiency of substrate processing), or mixed (apparently changes both *k_cat_* and *K_m_*) depending on their kinetic behavior. This kinetic behavior can be modeled as the inhibitor binding to different forms of the enzyme, as seen in Figure 2.^23^,^24^

In practice, most inhibitors intended as nuclear imaging agents are competitive inhibitors, which can be conceptually thought of as binding to the active site and blocking substrate binding and subsequently catalysis. Therefore, substrate, product, or transition state analogues are often the starting point for inhibitor design. Reversible inhibitors should have either *K_i_* or IC_50_ values measured. *K_i_* is the equilibrium constant for binding of the inhibitor, which is the ratio of the rate constant for enzyme–inhibitor dissociation divided by the ratio of enzyme–inhibitor association (*k_off_*/*k_on_*). IC_50_ is the concentration at which enzyme activity is reduced by 50% under given assay conditions. The advantage of measuring a *K_i_* value is that it is independent of enzyme and substrate concentrations, so values measured in different laboratories, different cell lines, and under different conditions can be directly compared. As well, careful measurements of a *K_i_* value will help identify and properly characterize potentially slow- and tight-binding reversible inhibitors.^25,26^ Such incredibly potent inhibitors may have a biological effect even at tracer levels, similar to some of the most potent imaging agents for the opioid receptors.^27^ The major advantage of measuring an IC_50_ value is that measurements are easier and faster (requiring only approximately 15%-20% of the number of data points^28^) which can facilitate a quick determination of relative inhibitor potency when evaluating a number of potential inhibitors as imaging tracers or when quickly validating a known substrate that has been modified for molecular imaging (eg, radiolabeled peptides, drugs).

Ideal reversible inhibitors used as imaging agents should have low *K_i_* or IC_50_ values, since those potent inhibitors will concentrate more readily in sites of enzyme expression. However, there is a practical lower limit for the *K_i_* value (*k_off_*/*k_on_*) of inhibitor intended for imaging enzyme distribution. Since *k_on_* is limited by the rate of diffusion (∼10^8^ M^−1^s^−1^),^29^ extremely strong inhibitors must derive their potency from reducing the rate of *k_off_*. In practice, this means that inhibitors with *K_i_* < ∼10 nM are members of a subclass called tight-binding inhibitors^25,26^ and will not rapidly equilibrate with their target enzyme. The resulting images will not reflect enzyme expression levels, because tracer washout does not occur due to slow off rates and therefore does not reach an equilibrium distribution as kinetic modeling for a reversible inhibitor demands. Indeed, a very potent inhibitor begins to behave kinetically more like an irreversible inhibitor (discussed below) rather than a reversible inhibitor leading to images that do not reflect differences in enzyme distribution within a given tissue or organ.^14^ This information is needed for each individual radiotracer used for imaging different enzymes, as correct classification (eg, reversible vs irreversible vs tight-binding reversible) is needed to perform correct kinetic modeling and properly interpret PET imaging data. The dissociation of the tight-binding inhibitor methotrexate from dihydrofolate reductase demonstrates how slow off rates can affect the lifetime of the inhibitor–enzyme complex: The half-life for dissociation of the enzyme–inhibitor complex, as estimated by dialysis experiments, is greater than 6 days.^30^ If such an inhibitor were to be radiolabeled and monitored using PET/SPECT, kinetically modeling assuming reversible inhibition (with rapid enzyme–inhibitor association–dissociation rates) would be clearly inappropriate.

### Irreversible Inhibitors

**Figure 3.**
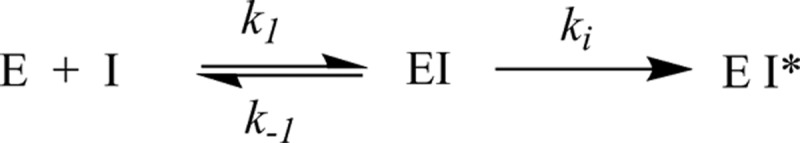
Kinetic scheme for irreversible inhibition of an enzyme.

Irreversible inhibitors reduce enzyme activity through formation of a stable covalent bond between the inhibitor and the target enzyme. These are most often mechanism-based inactivators, which are substrate analogues that are activated by the enzyme’s catalytic machinery to reveal a reactive moiety (most often a latent electrophile) that subsequently covalently binds to an amino acid residue in the active site resulting in a loss of activity. Inhibition can occur through covalent modification of a key catalytic residue (eg, alkylation of a carboxylate or amine sidechain) or by physically blocking access to the active site. Kinetically, this process is analogous to processing of a substrate by the enzyme and can be represented by Equation 2, which is described in Figure 3.^31^

2 $$v=\frac{v_{\text{max}}\left[I\right]}{K_{i}+\left[I\right]}.$$

In this equation, *k_i_* (which is analogous to *k_cat_* for a substrate) is the rate constant for the reaction that generates the inactivated form of the enzyme (EI*) once the enzyme is saturated by the irreversible inhibitor. A *k_i_* value is obtained by dividing the maximum rate of inactivation (*V*_max_ for the inactivation reaction) by the total enzyme concentration. *K_i_* is the concentration at which the inactivation reaction proceeds at 50% of the maximum velocity (analogous to *K_m_* for a substrate). The ratio of *k_i_*/*K_i_* is the second-order rate constant for the reaction between free enzyme and free inhibitor to form the inactivated form of the enzyme. Note that the *K_i_* value for an irreversible inhibitor is conceptually very different from the *K_i_* value for a reversible inhibitor, meaning it is essential to clearly represent the class of inhibitor when reporting *K_i_* values.

Ideally, irreversible inhibitors should have high *k_i_* values that indicate a rapid reaction between inhibitor and enzyme forming the covalent inhibitor–enzyme complex and low *K_i_* values which generally reflect strong noncovalent preassociation of the inhibitor and the enzyme prior to the inactivation reaction. Inhibitors with large *k_i_*/*K_i_* values bind to and react quickly with their target enzymes. Because the action of an irreversible inhibitor is time dependent,^31^ measuring IC_50_ values is inappropriate since IC_50_ values are highly dependent on the substrate concentration and incubation time within an experiment. However, as mentioned above, there is a practical limit to irreversible inhibitor efficiency when used for radiotracer imaging. Irreversible inhibitors whose efficiency is above a certain limit (which is unique for each enzyme) produce images that actually reflect blood flow rather than enzyme activity. This situation has notably been observed for extremely rapid irreversible inhibitors of both acetylcholinesterase (AChE)^32,33^ (discussed below) and MAO.^15^ For irreversible inhibitors which are not so rapid as to be limited by blood flow, the *k_i_*/*K_i_* value should reflect efficiency under physiological concentrations of enzyme and tracer levels of imaging agent. Often, radiotracer derivatives having varying *k_i_*/*K_i_* values must be evaluated in vivo accompanied by kinetic modeling to account for blood flow to determine the ideal inactivation efficiency required for quantitative imaging experiments of a particular target.

## Imaging Enzyme Activity in the Brain

Some hydrolytic enzymes that are of interest for imaging with PET/SPECT are located in the brain, meaning a small molecule tracer must access the central nervous system (CNS). All imaging agents, both in the CNS and in peripheral systems, generally need to be selective for their target, reasonably tight-binding to their target, moderately hydrophilic (to avoid widespread nonspecific binding, especially to lipids), reasonably resistant to metabolism, easily synthesized and radiolabeled, and safe at tracer levels (which is largely dependent on the specific activity of the injected tracer). Additionally, to image targets in the brain, a molecule must also be able to cross the BBB. To cross the BBB, a molecule generally must be relatively small (<500 g/mol and <80 Å cross-sectional area), moderately hydrophobic (log *D* 2.0 − 3.5), neutral, and have few hydrogen-bond donor and acceptor sites. Additionally, a probe must not be a substrate for efflux transporters such as P-glycoprotein (PgP) or breast cancer–resistance protein (Bcrp).^34-36^ Although progress continues to be made in the development of imaging agents for enzymes in the CNS, probe design remains an ongoing challenge.^37^

## EC 3.1: Esterases

### Acetylcholinesterase and Butyrylcholinesterase

Acetylcholinesterase plays a critical role in nerve signaling, as it is responsible for the postsynaptic hydrolysis of the neurotransmitter acetylcholine following activation of the postsynaptic neuron. Molecular imaging of AChE is of interest to help clarify the role of the enzyme in Alzheimer disease progression, in particular.^34,38^ Imaging of AChE has been well studied and an interested reader should consult one of the excellent reviews on recent progress in radionuclide-based molecular imaging for AChE.^38-40^ Since publication of those reviews, several more articles on PET imaging of AChE have appeared.^41-47^

More recently, butyrylcholinesterase (BChE) was identified as a potential imaging target since it also can play a role in acetylcholine metabolism, especially in patients with neurodegenerative diseases such as Alzheimer’s.^48^ Specific imaging of BChE is a new area of interest, and recently agents specifically targeting BChE have been reported.^49,50^

### Lipase

**Figure 4.**
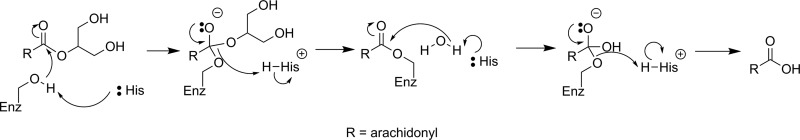
Mechanism for hydrolysis of 2-AG by MAGL.^51^ 2-AG indicates 2-arachidonoylglycerol; MAGL, monoacylglycerol lipase.

Monoacylglycerol lipase (MAGL) is a serine hydrolase which catalyzes the postsynaptic hydrolysis of 2-arachidonoylglycerol (2-AG; see Figure 4), which is an endocannabinoid neurotransmitter.^52^ 2-Arachidonoylglycerol is synthesized by the postsynaptic neuron and diffuses to the presynaptic neuron where it activates cannabinoid receptors, leading to a reduction in presynaptic neurotransmitter release. The hydrolytic activity of MAGL is thought to be responsible for terminating approximately 85% of the signal from 2-AG.^53^ Since problems with the endocannabinoid system have been suggested to play a role in a number of neurological diseases such as addiction, pain, anxiety, and schizophrenia, small molecule tools to modulate endocannabinoid activity without unwanted psychoactive effects are desirable both as therapeutics and probes of endocannabinoid function.^54^ For these reasons, MAGL has recently been identified as a target of interest for imaging with PET/SPECT.

**Figure 5.**
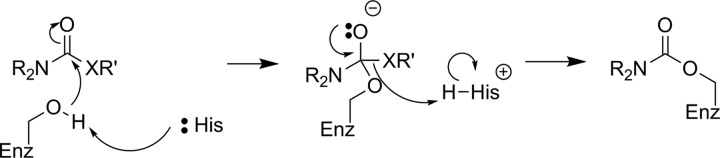
Reaction of MAGL with carbamate-based (X = O) or urea-based (X = NH) irreversible inhibitors to form a stable enzyme–inhibitor complex. MAGL indicates monoacylglycerol lipase.

**Figure 6.**
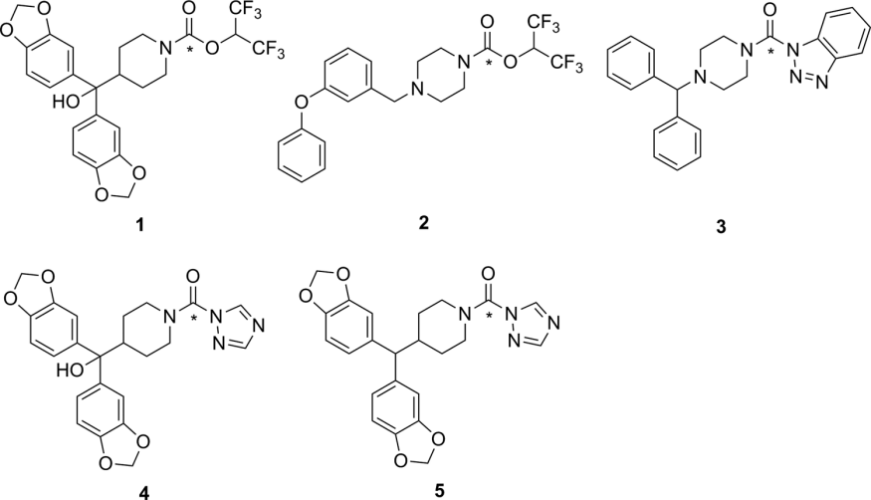
Carbamates (**1** and **2**) and ureas (**3**-**5**) tested as irreversible inhibitors of MAGL. *^11^C. MAGL indicates monoacylglycerol lipase.

**Figure 7.**
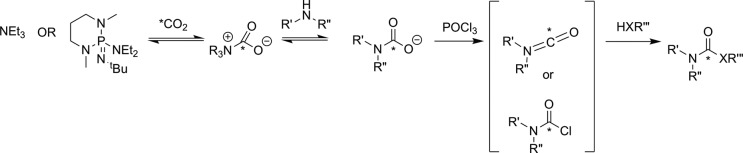
Reaction of [^11^C]CO_2_ to form carbamates (X = O) or ureas (X = NR). *^11^C.^56^

The first attempt to develop a PET imaging agent for MAGL was by Wilson and coworkers, who prepared a total of 12 putative irreversible inhibitors for MAGL (7 known compounds and 5 novel molecules).^55^ These irreversible inhibitors were designed to tag the catalytic nucleophilic serine of MAGL through reaction with an activated carbamate or urea moiety present in the inhibitor to form a very stable covalent–enzyme intermediate (see Figure 5). To estimate inhibitor potency and specificity, IC_50_ values were measured for all 12 compounds as inhibitors of both MAGL and fatty acid amide hydrolase (FAAH), which is a related enzyme responsible for terminating endocannabinoid signaling through hydrolysis of anandamide, as discussed below. A further complicating factor in development of an imaging agent for MAGL in the brain is the modest level of MAGL activity in the blood that could potentially lead to high background signal by nontarget tracer binding. Five of the inhibitors (**1**-**5**, Figure 6) were selected as interesting leads and radiolabeled with ^11^C at the carbonyl in the carbamate or urea functionality. The radiochemistry to prepare [^11^C]**1**-**5** relies on the relatively recent development of [^11^C]CO_2_ fixation and activation toward reaction with alcohols, phenols, and amines using POCl_3_.^56^ Capture of [^11^C]CO_2_ with an amine such as diazabicyclo[5.4.0]undec-7-ene, triethylamine, or 2-*tert*-butylimino-2-diethylamino-1,3-dimethyl-perhydro-1,3,2-diazaphosphorine followed by activation with POCl_3_ and substitution with an appropriate nucleophile (Figure 7) led to the formation of [^11^C]**1**-**5**. Ex vivo analysis and autoradiography in mice and rats examined the biodistribution and brain uptake of all 5 compounds, and specificity was tested by preinjection of an unlabeled version of the inhibitor to measure the decrease in tracer accumulation (blocking study). All 5 inhibitors showed limited and nonspecific uptake in the brain, although [^11^C]**5** showed a meaningful reduction of radioactivity upon pharmacological challenge with the corresponding unlabeled analogue **5**. The authors concluded that the limited BBB permeability, coupled with the rapid metabolism of blood-bound MAGL, made imaging of MAGL in the brain a very difficult target using any of the tracers tested.^55^

**Figure 8.**
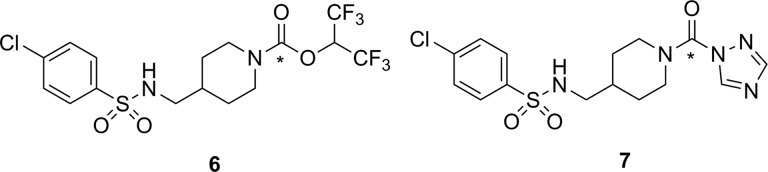
Recently reported ^11^C-labeled irreversible inhibitors for MAGL. *^11^C. MAGL indicates monoacylglycerol lipase.

Recently, 2 independent radiosyntheses were reported for [^11^C]SAR127303 (**6**, Figure 8), which is a carbamate-containing irreversible inhibitor of MAGL.^57,58^ The unlabeled analogue of **6** (ie, **6**) has been previously prepared and identified as a potent and selective inhibitor of MAGL, albeit with reported side effects on memory and learning that preclude its therapeutic use.^59^ However, such undesirable drug side effects were deemed unlikely to be a problem for use of [^11^C]**6** as an imaging agent injected at tracer levels. Hooker and coworkers first described the radiosynthesis of [^11^C]**6** using [^11^C]CO_2_ fixation, along with some initial biological and imaging data in rats. The PET scanning of rats treated with [^11^C]**6** showed rapid uptake in the brain with regions of heterogeneity indicating binding to a target in the brain. Blocking studies by preinjection with **6** showed reasonable selectivity, with only ∼30% of the observed signal being attributed to nonspecific binding of the tracer, assumed to be the lipid-rich white matter. Following injection of [^11^C]**6**, injection of its nonradioactive analogue **6** was unable to displace the radiotracer from MAGL since no decrease was observed in the PET signal, demonstrating that a covalent and irreversible reaction took place between [^11^C]**6** and MAGL.^57^ Shortly thereafter, Liang and coworkers reported a slightly different (albeit comparable) radiosynthesis for [^11^C]**6**. They also described the preparation of a variety of derivatives designed to study structure–activity relationships in different elements of the inhibitor (aryl group, linker, and the leaving group). On the basis of in vitro selectivity, potency, and likely BBB penetrance (predicted by lipophilicity and molecular size), **6** and a 1,2,4-triazole derivative **7** were selected for radiolabeling and further evaluation. Ex vivo biodistribution studies in mice, followed by imaging studies in rats and a preliminary scan in a rhesus monkey were performed. Injection of [^11^C]**7** into rats produced heterogeneity in brain regions of interest, although blocking studies with unlabeled **7** showed only a modest reduction of signal.^58^ The imaging data from injection of [^11^C]**6** into rats was comparable to the earlier report,^57^ and initial data from scanning in monkey brain were encouraging for using [^11^C]**6** as the lead compound for development of a clinical tracer.^58^

### Phosphodiesterases

Cyclic nucleotide phosphodiesterase (PDE) enzymes are a family of 11 enzymes that play an important role in termination of cellular signaling by hydrolyzing the secondary messenger molecules cyclic adenosine monophosphate and cyclic guanosine monophosphate. Some members of this family of enzymes (PDEs 2, 4, 5, 7, and 10) have been identified as potential targets for PET imaging, especially in the brain. There has been a substantial amount of work in the preparation and testing of potential PET/SPECT imaging agents for PDEs, which have recently been covered in 2 excellent and comprehensive reviews covering developments prior to 2012^60^ and between 2012 and 2016.^61^

### Sulfatases

**Figure 9.**
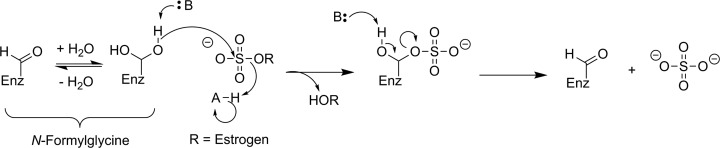
Proposed mechanism for STS hydrolysis of an estrogen-3-sulfate.^62^ STS indicates steroid sulfatase.

Steroid sulfatase (STS; estrone sulfatase) has been identified as a potential target for imaging with PET/SPECT. Increased STS activity has been demonstrated in some hormone-dependent breast cancers: The enzyme cleaves sulfate from biologically inactive estrogen-3-sulfates (estrone-3-sulfate, estradiol-3-sulfate, or estriol-3-sulfate) to release the desulfonated estrogen (see Figure 9), which in turn increases tumor growth by activating the estrogen receptor. The aim of developing an imaging agent for STS is to visualize and differentiate breast cancer subtypes. Given that other targets such as estrogen receptors already allow PET/SPECT imaging of breast cancer subtypes,^63^ there is not a pressing need for development of an imaging agent targeting STS.

**Figure 10.**
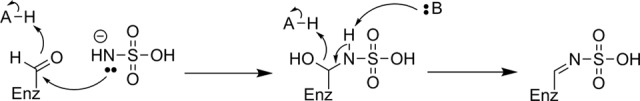
Proposed mechanism of irreversible inhibition of STS by a sulfonamide through imine *N*-sulfate formation with the active-site formylglycine residue.^62^ STS indicates steroid sulfatase.

**Figure 11.**
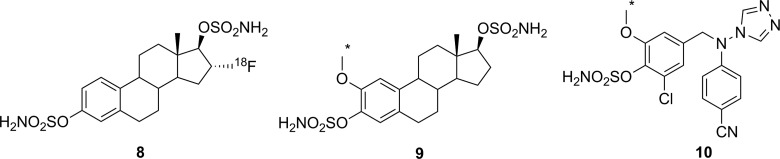
Irreversible inhibitors prepared as potential PET imaging agents for STS. *^11^C. PET indicates positron emission tomography; STS, steroid sulfatase.

There have been a few attempts to image STS activity, though no successes have been reported. All of the reported radiolabeled probes for STS are irreversible inhibitors with an aryl sulfamate moiety. While the mechanism for irreversible inhibition of STS with an aryl sulfamate has not been definitively proven, these inhibitors have been proposed to function through formation of an imine *N*-sulfate with the catalytic formylglycine residue (Figure 10).^62,64,65^ However, carbonic anhydrase (CA, EC 4.2.1.1) is also sensitive to inhibition with aryl sulfamates, meaning that selectivity between these 2 enzymes is an important consideration for development of a nuclear imaging agent.^65^ Derivative [^18^F]**8** (Figure 11) was the first radioactive compound prepared as a potential PET imaging agents of STS. Owing to their thermal instability, it was necessary to install the sulfate and sulfamate functionalities after the high-temperature radiofluorination with ^18^F-fluoride and removal of protecting groups. The unlabeled analogue **8** was shown to be a good inhibitor of both STS and CA, and biodistribution studies in rats, mice-bearing tumor xenografts, and piglets with [^18^F]**8** demonstrated that the radioactive signal came primarily from blood.^66^ Persistent signal in the blood is presumably a consequence of reaction between [^18^F]**8** and CA found in red blood cells, which is known to be highly expressed in erythrocytes.^65^ The related ^11^C-labeled compound [^11^C]**9** was also prepared to test whether introduction of an *ortho*-methoxy group would confer selectivity for STS over CA. Measured IC_50_ values with **9** suggested a 10:1 selectivity for STS over CA,^67^ although whether in vitro IC_50_ values can be used to adequately evaluate selectivity of an irreversible inhibitor whose action is dependent on the kinetics of inactivation is questionable. Since no in vivo imaging studies were reported,^67^ it remains unclear whether the reported selectivity will result in increased tumor accumulation through reaction with STS, compared to remaining bound to the blood through reaction with CA. Finally, a dual aromatase/STS inhibitor [^11^C]**10** has been ^11^C-labeled using [^11^C]CH_3_I as one of a series of compounds intended for breast cancer imaging. Although a good inhibitor of STS as judged by the in vitro IC_50_ value using **10**, no biological data have been reported to date.^68,69^ In summary, while there have been efforts at designing STS irreversible inhibitors as PET imaging agents, there has been very little biological data reported and no major successes.

## EC 3.2: Glycosylases

### Glycosidases

Glycoside hydrolases are a large family of enzymes responsible for hydrolytic cleavage of 1 or more sugar residues from a variety of biomolecules, including oligosaccharides or polysaccharides, peptides or proteins, and lipids. Glycosidases have been shown to play important roles in a number of diseases, including diabetes,^70^ Parkinson disease,^71^ cancer,^72^ and metabolic disorders such as Gaucher disease.^73^ However, despite their biological importance, only moderate progress has been made in developing PET/SPECT imaging agents for glycosidases, despite a number of attempts using different approaches.

**Figure 12.**
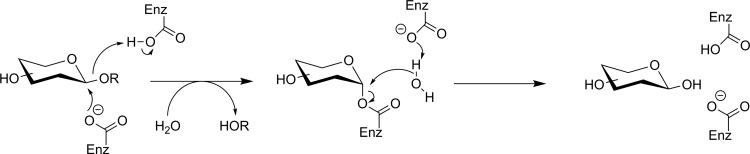
General retaining β-glycosidase mechanism.^74^

Three retaining β-glycosidases have frequently been targeted as potential nuclear imaging markers: *Escherichia coli* β-galactosidase (lacZ), human lysosomal β-glucuronidase (GUS), and human β-glucocerebrosidase (acid β-glucosidase, GBA1). Figure 12 shows a generic retaining β-glycosidase mechanism. LacZ is an enzyme of interest owing to its frequent use as a reporter gene; an imaging agent could be used to identify expression of a gene of interest in a whole organism in a noninvasive way. GUS, normally restricted to the lysosome, is overexpressed in the extracellular tumor microenvironment and is a potential imaging target for tumor localization. GBA1 is also a lysosomal enzyme whose deficiency leads to Gaucher disease.^75^ More recently, mutations in the gene encoding for GBA1 have also been identified as the single largest genetic risk factor for the development of Parkinson disease, and low levels of GBA1 enzyme activity found in sporadic, early, and late Parkinson patients make it a potential diagnostic and/or therapeutic target.^76^

**Figure 13.**
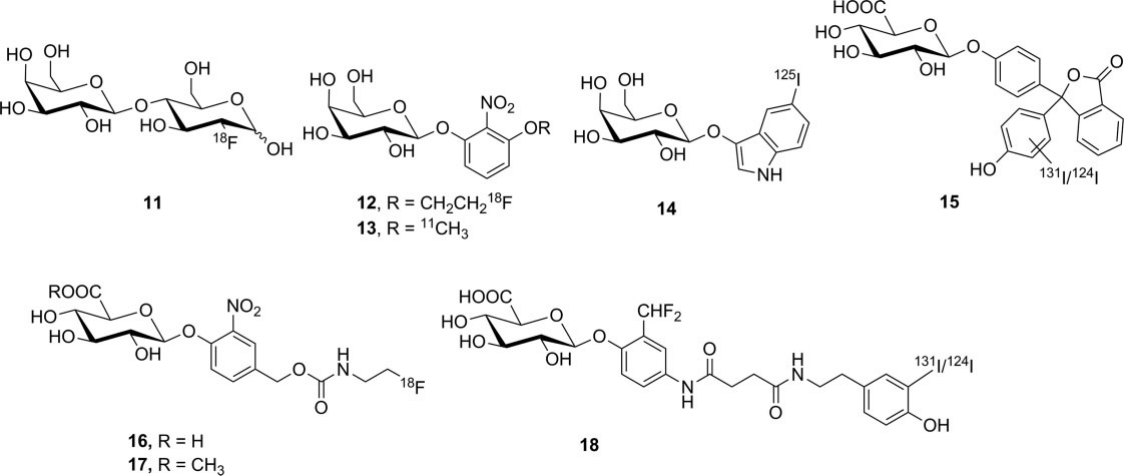
Compounds tested as substrate-based nuclear imaging agents for lacZ (**11**-**14**) and GUS (**15**-**18**). GUS indicates β-glucuronidase.

**Figure 14.**
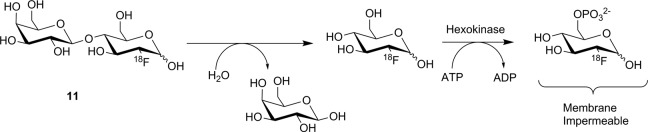
Proposed mechanism for radiotracer accumulation following hydrolysis of **11** by lacZ.^77^

Four compounds (**11**-**14**, Figure 13) have been prepared as potential substrate-based nuclear imaging agents for lacZ. The first substrate tested was 2′-deoxy-2′-[^18^F]fluorolactose ([^18^F]**11**). This agent was designed to image lacZ expression through cleavage of the β-galactosyl residue to release free [^18^F]FDG, as seen in Figure 14. Following hydrolysis, [^18^F]FDG was intended to be metabolically trapped by hexokinase after phosphorylation. This putative substrate was prepared by the enzyme-catalyzed transfer of a galactose moiety onto [^18^F]FDG, and despite what the authors described as poor radiochemical yield (3.4%, decay corrected), sufficient labeled material was prepared to enable preliminary testing. Incubation with purified enzyme in vitro showed that **11** was a competent substrate for lacZ, although no quantitative kinetics were reported. Injection of the tracer into mice showed no uptake in groups of mice that were either positive or negative for expression of lacZ, which the authors attributed to membrane impermeability of [^18^F]**11**.^77^ Two unrelated substrates, **12** and **13**, were also prepared, which are derivatives of the common chromogenic substrate *ortho*-nitrophenyl β-D-galactopyranoside (oPNG). These derivatives differed only in the presence of the –OR moiety, which was used to affix the radiolabel. Measuring % activity relative to oPNG suggested both **12** and **13** were good substrates for lacZ in vitro and had good metabolic stability in mice (showing little turnover by the endogenous murine β-galactosidase activity). However, PET imaging in mice showed that neither [^18^F]**12** nor [^11^C]**13** crossed the BBB, limiting utility of these reagents as potential imaging agents for gene expression in the brain. In the peripheral tissues, accumulation was observed in the kidneys and liver but not in xenograft tumors expressing lacZ. This observed distribution suggested that if the substrate was hydrolyzed by lacZ, the radiolabeled aromatic phenol freely diffused out of cells and was not retained at the site of activity (ie, no tumor retention).^78^ The final substrate-based agent for measuring lacZ activity using SPECT was [^125^I]**14**. This compound was designed to immobilize at the site of enzyme activity through oxidative dimerization of the released [^125^I]iodoindoxyl aglycone^79^ in a reaction analogous to the spontaneous dimerization of the 5-bromo-4-chloro-3-hydroxyindole aglycone released by hydrolysis of X-gal.^80^ Tests using the nonradioactive analogue showed the compound could cross cell membranes and immobilize at 0.5 mM, as judged by accumulation of a blue precipitate. However, tests in mice with [^125^I]**14** showed no uptake, only renal clearance. Although intratumoral injection did demonstrate somewhat slower clearance from lacZ-expressing xenograft tumors compared to tumors that did not express lacZ,^79^ this approach for immobilizing the radiolabel seems unlikely to be successful. The extremely low concentrations of indoxyl produced at tracer levels mean the rate of the dimerization reaction is likely to be incredibly slow relative to the rate of diffusion out of the cell, leading to a loss of radioactive signal and no detection.

**Figure 15.**
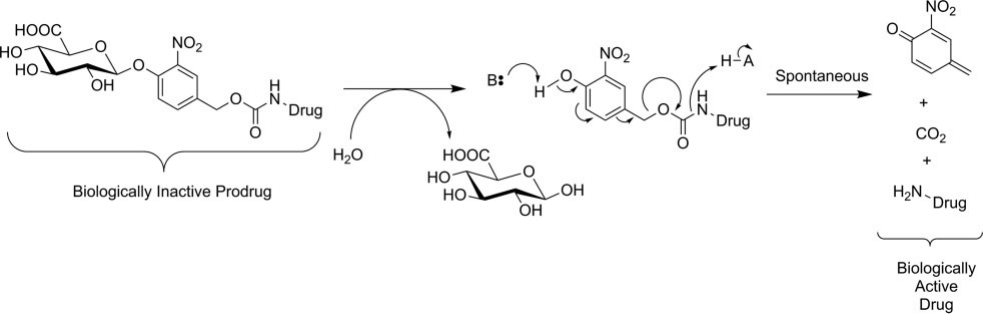
Activation of a glucuronide prodrug conjugate by GUS. The active form of the drug is released through spontaneous decomposition of the self-immolative linker following enzyme-mediated hydrolysis.^84^ GUS indicates β-glucuronidase.

**Figure 16.**
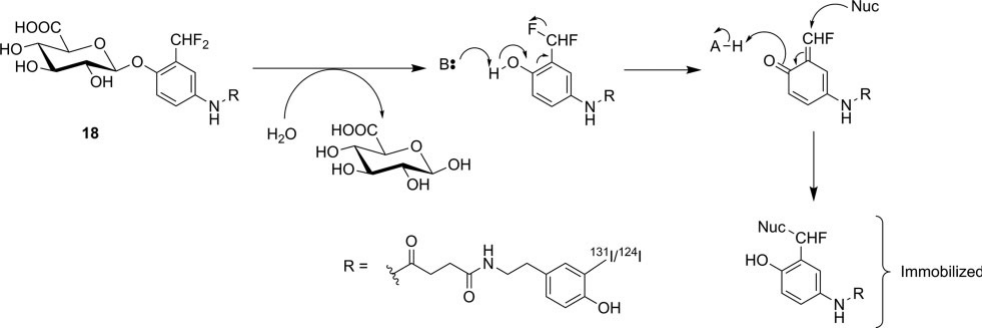
Hydrolysis of **18** by GUS, followed by proposed nucleophilic addition to a cell-based nucleophile to immobilize the tracer-labeled aglycone.^86^ GUS indicates β-glucuronidase.

Efforts to image GUS activity using metabolic trapping following hydrolysis of a substrate have been more successful. Since GUS activity is found in the extracellular tumor microenvironment,^81^ efficient membrane permeability is not a concern during probe design. Indeed, having a membrane impermeable tracer would avoid ubiquitous low-level GUS activity in the lysosome and high normal tissue uptake, so only tumor tissues with high extracellular GUS activity will be detected. However, radiotracer substrates intended for imaging of GUS activity also have the drawback that glucuronylation is a common in vivo modification of xenobiotics to increase solubility prior to renal excretion, meaning that glucuronylated tracers are highly likely to accumulate in the kidneys and bladder. Compound **15** was the first tracer developed, with a hydrophobic [^124^I]iodophenolphthalein aglycone for PET imaging. The aglycone was designed to associate with hydrophobic components of the cell membrane at the site of hydrolysis. No in vitro enzyme kinetics for nonradioactive [^127^I]**15** as a substrate for GUS were reported, and instead an assay measuring precipitation of the dark reddish dye around cells engineered to express GUS on their surface was used to show that [^127^I]**15** was processed by the enzyme. The precipitation reaction could be blocked by treatment with saccharic acid 1,4-lactone, a known inhibitor of GUS. **15** was radiolabeled with ^131^I for biodistribution studies using gamma scintigraphy and with ^124^I for microPET studies in mice. ^124^I-labeled **15** was injected into xenograft mice bearing 2 tumors, 1 of which did and 1 of which did not express GUS anchored on its cell surface. It was observed that tracer uptake was ∼3.5-fold increased in tumors-expressing GUS, specific uptake which could be blocked by prior injection of saccharic acid 1,4-lactone. Substantial signal was also detected in the gallbladder, liver, and intestines, indicating that tracer metabolism and excretion were occurring.^82^ Shortly thereafter, [^18^F]**16** was reported as a substrate-based imaging agent for GUS activity.^83^ The design of **16** was inspired by progress made in prodrug therapy for specifically targeting GUS-expressing tumors with anticancer drugs. In this approach, the cytotoxic drug is rendered biologically inactive through conjugation to a glucuronyl residue through a self-immolative linker. Activation of the linker through GUS-mediated hydrolysis of the glucuronyl residue leads to spontaneous decomposition of the linker to form a quinone methide, CO_2(g)_, and the free drug (Figure 15).^84^ Instead of a drug, tracer **16** incorporated an [^18^F]fluoroethylamine group that was designed to diffuse into cells and accept a proton in acidic organelles such as lysosomes. The resulting cation should remain metabolically trapped, as the charged tracer is membrane impermeable. One benefit to this compound is that production of a 4-hydroxymethyl-2-nitrophenol chromophore from the decomposition of the linker could be monitored using UV-visible spectrophotometry, meaning that complete in vitro kinetics could be measured for **16** as a substrate for *E coli* GUS and bovine GUS. **16** was a good substrate for both bacterial GUS (*k_cat_*/*K_m_* = 24.6 × 10^6^ M^−1^·s^−1^) and bovine GUS (*k_cat_*/*K_m_* = 1.8 × 10^6^ M^−1^·s^−1^), and testing in cellulo with exogenously added bacterial GUS showed a 6-fold increase in radioactivity inside the cells, demonstrating that the [^18^F]fluoroethylamine was accumulating in nearby cells as intended. Preliminary in vivo studies with xenograft mice showed clearance primarily through the kidneys and a 2-fold increase in tracer uptake in GUS-expressing tumors relative to tumors that did not express GUS. The authors noted that while [^18^F]**16** was very stable in plasma, the rapid clearance by the kidneys hindered tumor uptake.^83^ A follow-up study by the same group attempted to reduce renal clearance by preparation of a methyl ester analogue ([^18^F]**17**) of the tracer to reduce recognition by transporters for glucuronylated xenobiotics. However, they observed that endogenous esterases in plasma rapidly hydrolyzed the methyl ester and [^18^F]**17** showed no improvement in tracer kinetics relative to the free carboxylic acid [^18^F]**16**.^85^ Finally, **18** was also recently reported as a nuclear imaging agent for GUS. Following hydrolysis of glucuronyl residue, **18** decomposes through spontaneous intramolecular loss of a fluoride to form an *ortho*-quinone methide. The quinone methide is a well-established electrophile and was expected to immobilize near the site of enzyme activity through formation of a covalent bond on nucleophilic amino acid residues on nearby proteins (Figure 16). The authors suggested that the nucleophile belonged to nearby membrane-bound proteins,^86^ although no specific evidence for this assumption was reported. Indeed, since the effects of **18** on GUS activity as a function of incubation time were not reported, it is unclear whether **18** is a true substrate or a “repeat attack” inhibitor of GUS. Such inhibitory behavior is a possibility since the reactive *ortho*-quinone methide conceivably could react with a nucleophile in the GUS enzyme, similar to what has been previously observed for a bacterial glucosidase treated with a quinone methide-forming natural product.^87^ The same group has also previously prepared near-infrared^88^ and fluorescent^89^ probes for GUS using a similar scaffold, and those compounds proved helpful while testing **18** as a potential PET probe for GUS. Although no enzyme kinetic values were reported, cell studies with a nonradioactive fluorescein-labeled analogue of [^127^I]**18** showed that cells expressing GUS on their surface were irreversibly fluorescently labeled. This labeling happened only in the presence of GUS and could be blocked by the presence of a known GUS inhibitor saccharic acid 1,4-lactone. In cells, the probe showed no toxicity at concentrations up to 100 μg/mL, suggesting tracer levels of the probe would not produce toxic levels of quinone methide in vivo. Injection into live mice also showed no signs of hematological abnormalities nor liver injury. Injection of ^124^I-labeled **18** into xenograft mice bearing tumors showed an impressive uptake ratio of up to 141-fold at 20 hours by PET scanning for tumors expressing GUS on their cell surface compared to tumors with no GUS on the cell surface. However, substantial PET signal was also observed in the abdomen. Ex vivo biodistribution studies (using ^131^I-labeled **18**) showed considerable uptake in the liver, bladder, kidney, stomach, and urine, suggesting that metabolism of the tracer was occurring rapidly.^86^ In summary, substrate-based imaging probes for GUS have made considerable progress, and the metabolic trapping strategies employed have been well designed and generally successful. Whether such probes can overcome the almost inevitable challenge of metabolism and excretion owing to the glucuronyl “tag” remains to be seen.

**Figure 17.**
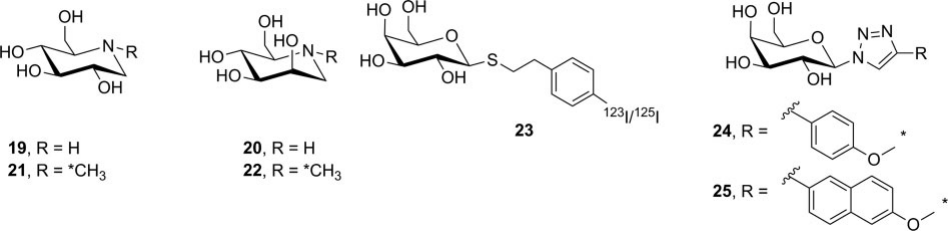
Reversible inhibitors prepared as potential glycosidase imaging agents. *^11^C.

Radiolabeled reversible glycosidase inhibitors have also been prepared as potential PET/SPECT imaging probes. Nojirimycin (**19**, Figure 17) is a potent α-glucosidase inhibitor, and its epimer **20** was presumed, but at the time not proven, to be an α-mannosidase inhibitor. The ^11^C-labeled-*N*-methyl derivatives [^11^C]**21** and [^11^C]**22** were separately synthesized by reaction of [^11^C]CH_3_I with **19** and **20**, respectively. The goal with these tracers was to image glycoprotein synthesis in mice with xenograft tumors, since α-glucosidase activity (inhibited by **19**) and mannosidase activity (presumably inhibited by **20**) are both involved in glycan remodeling during glycoprotein synthesis. Ex vivo biodistribution analysis in mice revealed that uptake of [^11^C]**21** was primarily in the kidney, liver, and small intestine, with moderate amounts of tracer detected in tumor. Tracer accumulation in the brain was extremely low. Organ uptake could be blocked by pretreatment with **19**, suggesting that [^11^C]**21** was binding to a glycosidase target. In contrast, uptake of [^11^C]**22** was uniform and could not be blocked by pretreatment with **20**, suggesting that [^11^C]**22** was not binding to endogenous mannosidases.^90^ The lack of observed specificity and questions about the particular enzyme(s) being engaged by [^11^C]**21** are probably why such compounds have not been reported in further imaging studies.

To date, 3 reversible inhibitors targeting lacZ (**23**-**25**) have been prepared. The radiosynthesis of [^123^I/^125^I]**23** was described, as was its per-*O*-acetyl derivative (which should have better membrane permeability).^91^ Although no PET/SPECT studies were reported, ^125^I-labeled **23** was studied in mice using planar gamma scintigraphy. Although the tracer was metabolically stable, uptake in xenograft mice bearing lacZ-expressing tumors appeared to be more sensitive to blood flow than lacZ expression.^92^ Additionally, Bormans and coworkers reported the synthesis and evaluation of 2 ^11^C-labeled triazole derivatives (**24** and **25**) as potential PET imaging agents for lacZ. While triazoles are known to inhibit β-galactosidase activity,^93^ **24** and **25** were only shown to inhibit oPNG hydrolysis by lacZ at high levels, making it impossible to judge whether this class of inhibitor is sufficiently potent to effectively image lacZ in vivo. Nonetheless, both ^11^C-labeled tracers were separately injected into mice and ex vivo biodistribution studies were performed which showed no appreciable tumor uptake. Subsequent work in cellulo showed that neither compound appeared to cross the cell membrane, thereby explaining the lack of observed organ accumulation.^93^ This is yet another example of a new potential molecular imaging agent requiring more detailed in vitro analysis prior to selecting the most promising candidates.

**Figure 18.**
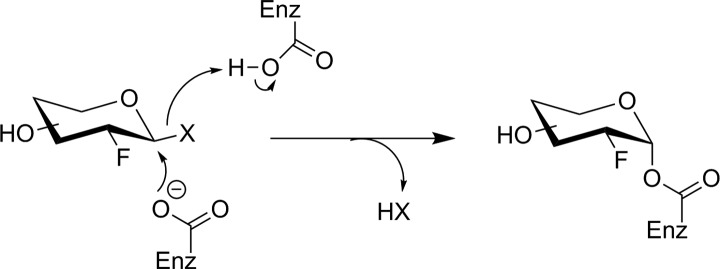
Irreversible inhibition of a retaining β-glycosidase by an activated 2-deoxy-2-fluoro glycoside. X = fluorine or O-dinitrophenyl.^94^

**Figure 19.**
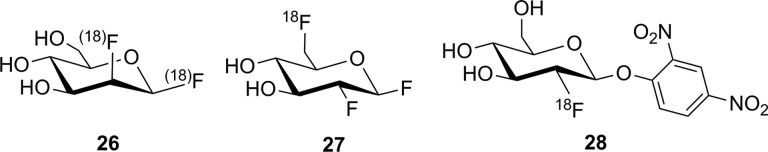
Irreversible inhibitors investigated as GBA1 imaging agents. Note that an individual molecule of [^18^F]**26** is nonspecifically radiolabeled at only 1 of 2 potential sites indicated with parentheses.

**Figure 20.**
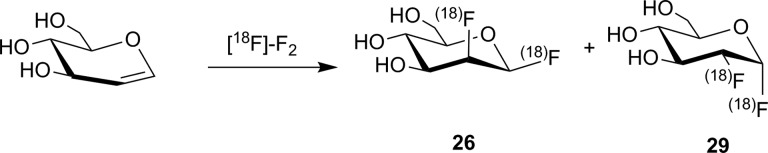
Reaction between glucal and [^18^F]F_2_ to form [^18^F]**26** and [^18^F]**29**. Note that an individual product molecule is nonspecifically radiolabeled at only 1 of 2 potential sites indicated with parentheses.^95^

The Withers group has shown that fluorosugars with good leaving groups (either fluoride or dinitrophenol) at their anomeric center are effective irreversible inhibitors for many retaining glycosidases.^94^ They inhibit enzyme activity by forming a long-lived fluorosugar–enzyme covalent conjugate (Figure 18). Fluorosugar inhibitors for retaining β-glucosidases typically have a 2-deoxy-2-fluoro modification, making them structurally similar to FDG. However, in only 1 instance (discussed below), has [^18^F]FDG served as the starting material for the synthesis of a fluorosugar inhibitor. [^18^F]2-Fluoro-2-deoxyglucose has not been the general starting material for fluorosugar inhibitors because the nonradioactive versions of such inhibitors are prepared through a nucleophilic substitution reaction at the anomeric center with a highly activated leaving group (to prepare the glycosyl fluoride) or a nucleophilic aromatic substitution reaction with the free sugar hemiacetal (to prepare the dinitrophenyl derivative). The time needed to selectively protect the other hydroxyl functional groups, perform the desired reaction at the anomeric center, and deprotect the other hydroxyl groups is incompatible with the rapid reactions required for short-lived isotope radiosynthesis. Therefore, early efforts to prepare fluorosugar glycosidase irreversible inhibitors ([^18^F]**26** and [^18^F]**27**, Figure 19) employed alternate radiosynthetic approaches. Compound **26** was prepared by reaction between [^18^F]F_2_ and glucal (Figure 20) to give a ∼2:1 mixture of β-*manno*- (**26**) and α-*gluco*-configured (**29**) [^18^F]difluoro sugars through *syn* addition of the ^18^F-labeled fluorine gas across the glucal alkene. Since an individual molecule of [^18^F]F_2_ is only radiolabeled on 1 of the 2 fluorine atoms, this procedure indiscriminately labels either the anomeric or 2-position of the sugar ring with the radioisotope. As a result, reaction with the enzyme produces inorganic [^18^F]fluoride by hydrolysis of the anomeric C–F bond in inhibitor molecules in which the ^18^F-label ends up attached to the anomeric center. [^18^F]**26** was purified and reacted with a model bacterial β-glucosidase in vitro.^95^ Subsequent biodistribution studies in rats using the nonradioactive **26** demonstrated that **26** was efficiently taken up in the brain and total mannosidase and glucosidase activities were inhibited. However, it was not determined which specific mannosidase(s) or glucosidase(s) reacted with the inhibitor.^96^ Presumably, because of this uncertain isozyme specificity, plus unavoidable release of [^18^F]fluoride upon glycosidase labeling, there have been no further reports of tracer [^18^F]**26** being tested as an imaging agent.

In an attempt to address some of these issues, a β-*gluco*-configured 6-[^18^F]fluoro analogue ([^18^F]**27**) was also prepared. The radiolabel was incorporated at the 6-position of the sugar ring for ease of synthetic access by S_N_2 displacement of a suitably protected 6-tosyl sugar derivative, despite the fact that this 6-deoxy-6-fluoro modification substantially reduced the efficiency of the inhibitor for the target enzyme GBA1. One problem with this approach was that the radiosynthesis was cumbersome (requiring silica gel purification) since it was necessary to completely remove the 6-hydroxy analogue by-product that contaminated the preparation from reaction with residual water, as the 6-hydroxy inhibitor was considerably more potent and would preferentially label the enzyme rather than tracer [^18^F]**27**. Although ^18^F-labeled **27** was successfully prepared, no in vitro labeling of enzyme was reported, nor any in vivo results.^97^ Given these challenges in preparing a radiolabeled fluorosugar glycosidase inhibitor, an alternate radiosynthetic approach was eventually devised. 2,4-dinitrofluorobenzene (Sanger’s reagent) was successfully reacted with [^18^F]FDG to form [^18^F]**28**. The resulting tracer was used to label Cerezyme,^98^ which is a recombinant and therapeutic form of human GBA1 used clinically to treat Gaucher disease. While reaction with [^18^F]**28** produced ^18^F-labeled Cerezyme in vitro and PET could be used to monitor the distribution of the injected therapeutic enzyme in mice, the fact that [^18^F]**28** is a global inhibitor for retaining β-glucosidase activity precludes its use for endogenous imaging of a specific β-glucosidase isozyme.

## EC 3.3: Ether Hydrolases

### Epoxide Hydrolase

**Figure 21.**
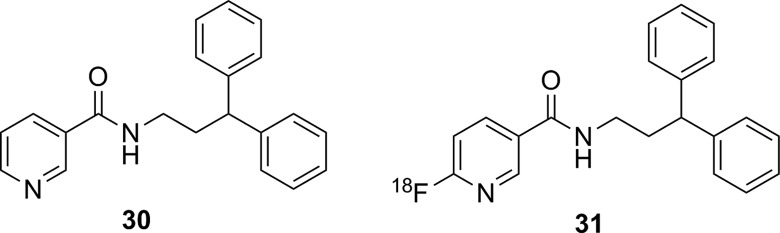
Known inhibitor for soluble epoxide hydrolase (**30**), and ^18^F-labeled derivative (**31**) used as a PET imaging agent. PET indicates positron emission tomography;

Recently, the development of a potential imaging agent for soluble epoxide hydrolase (sEH) has been reported.^99^ This enzyme catalyzes the degradation of epoxyeicosatrienoic acids, which are important signaling molecules for controlling blood flow in the brain. Inhibitors for this enzyme such as **30** (Figure 21)^100^ have been proposed as potential therapeutics in conditions, such as atherosclerosis, hypertension, inflammation, stroke, diabetes, among others.^101^ A PET/SPECT imaging agent for sEH could be useful for assessing brain function in patients with a stroke or dementia. Compound [^18^F]**31**, an ^18^F-labeled derivative of **30**, was prepared as an imaging agent for sEH. Both IC_50_ and *K_i_* values were measured for nonradioactive **31** as an inhibitor of sEH, and these inhibition studies verified that the replacement of a C–H for C–F on the pyridine ring did not substantially impact inhibitor potency. ^18^F-labeled **31** was prepared in high specific activity and purity, injected into both mice and baboons, and studied using PET. In both animals, regions of specific brain uptake were observed. This uptake could be blocked by preinjection of **30**, demonstrating no detectable nonspecific binding of [^18^F]**31**. Metabolism studies in baboon showed the only metabolites of [^18^F]**31** were more hydrophilic, which together with the blocking studies supports the idea that PET signal in the brain reflected sEH distribution.^99^

## EC 3.4: Proteases

Given their wide variety of critical biological roles, proteases are understandably a family of enzymes that have received a great deal of attention in the development of PET/SPECT imaging agents. All of the proteases of interest that have been investigated as potential nuclear imaging targets have been well studied and also reviewed recently. As a consequence, the treatment of those topics here will be cursory and will instead direct the interested reader to appropriate review articles or reports recently published.

### Cathepsins

Cathepsins are proteases that are often (though not universally) lysosomal enzymes involved in catabolic degradation of cellular components. Their dysfunction has been linked to a large number of diseases, including cancer, Alzheimer disease, arthritis, stroke, and parasitic infections.^102^ The PET/SPECT probes for cathepsins have been recently reviewed,^103^ and only a few articles on nuclear imaging of cathepsins have appeared since.^104-106^

### Prostate-Specific Membrane Antigen (Glutamate Carboxypeptidase 2)

Prostate-specific membrane antigen (PSMA; glutamate carboxypeptidase 2) is a membrane-bound metalloprotease that is highly upregulated in cancerous prostate cells, making it a very attractive target for imaging of primary and metastatic tumors. A large number of PET/SPECT imaging agents for PSMA have been described, and many excellent reviews have been published.^107-109^ Currently, prospective imaging agents for PSMA have advanced to small-scale clinical studies, hopefully leading to large-scale studies to validate their use in the diagnosis of metastatic prostate cancer.^110^

### Caspases

Caspases (cysteine-dependent aspartic proteases) are a family of cysteine proteases that play a key role in apoptosis, or programmed cell death, and in inflammation. Although lacking specific discussion of PET/SPECT imaging agents, a well-written comprehensive review on general probes for caspases, especially focusing on optical imaging agents and inhibitor development, has recently been published.^111^ However, a recent review from 2015 has surveyed PET/SPECT imaging agents for caspases 3 and 7.^112^ Since publication of that review, there have been several articles published on nuclear imaging agents targeting caspases.^113-119^

### Matrix Metalloproteases

Matrix metalloproteases (MMPs) are a large family of Zn^2+^-dependent endopeptidases primarily responsible for degradation of a variety of proteins in the extracellular matrix. They are involved in a wide variety of important biological processes, including apoptosis, cell proliferation and migration, differentiation, and angiogenesis. Owing to their upregulation in many cancers, MMPs 2 and 9 (gelatinases) have been the enzymes of most interest. A summary of advances in the development of potential imaging agents for MMPs was last published in 2013,^120^ and since then, there have been other reports of inhibitors^121-132^ and substrates^133,134^ designed to image MMP expression and activity, respectively. However, despite the considerable efforts to design an imaging agent for MMPs, to date, no imaging agent has made it into human trials.

## EC 3.5: Hydrolases Acting on Nonpeptidic Carbon–Nitrogen Bonds

### Histone Deacetylase

**Figure 22.**
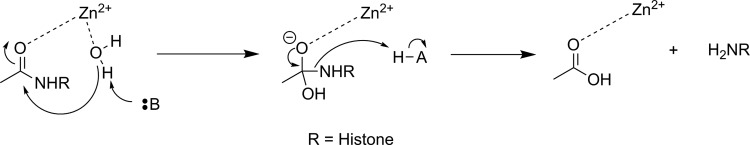
Simplified mechanism for a Zn^2+^-dependent HDAC.^136^ HDAC indicates histone deacetylase.

There are 4 classes of histone deacetylase (HDAC) enzyme: classes 1, 2, and 4 are zinc-dependent enzymes, while class 3 enzymes (also referred to as sirtuins) are NAD^+^ dependent.^135^ These enzymes are responsible for cleaving an acetyl moiety from the ∊-nitrogen on the side chain of a lysine in a histone protein. Figure 22 depicts a general reaction mechanism for a Zn^2+^-dependent (class 1, 2, or 4) HDAC. Protonation of the resulting free amine causes negatively charged DNA to wrap around the histone protein, thereby silencing expression of nearby genes, meaning HDACs are one mechanism for epigenetic control of gene expression.^135^ They have also been shown to act on a number of nonhistone proteins, meaning they have broader functions than just control of DNA expression.^137^

Altered HDAC levels have been detected in neurological degenerative diseases, such as Alzheimer disease, bipolar disorder, schizophrenia, major depressive disorder, stroke, epilepsy, and multiple sclerosis.^137^ Because of their role in epigenetic regulation, HDACs have also been linked to cancer.^135^ Class 1 and 2 HDAC activities have been particularly linked to disease and have been best studied. Class 1 enzymes are primarily found in the nucleus, while class 2 are much larger and can move between the nucleus and the cytoplasm. Class 2 can be further subdivided into 2a and 2b, which are both important for a variety of disorders, most notably various cancers.^135^ A tracer able to image variations in HDAC levels in both healthy and diseased individuals will be useful for better understanding the role of these enzymes in epigenetics, as well as studying the efficacy of potential therapeutics. It is important that such an imaging agent is able to cross the BBB, since many of the diseases of interest are brain disorders.

**Figure 23.**
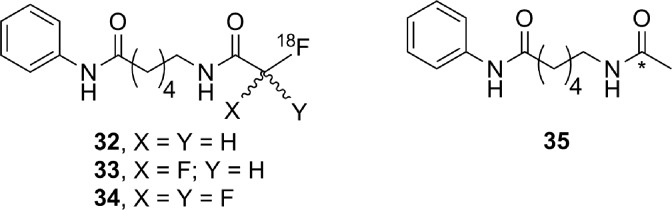
Potential substrate-based imaging agents for HDACs. *^11^C. HDACs indicates histone deacetylases.

Efforts to develop imaging agents for HDACs have focused on producing substrates and noncovalent inhibitors; no irreversible inhibitors have been described to date. The first attempt to image HDAC activity used 6-([^18^F]fluoroacetmido)-1-hexanoicanilide ([^18^F]FAHA or [^18^F]**32**, Figure 23) as a substrate for HDAC.^138^ The tracer was designed so that HDAC would release [^18^F]fluoroacetate, which would then accumulate intracellularly owing to the negative charge on the tracer. Studies with baboons on biodistribution, blockage by known HDAC inhibitors, and metabolic stability experiments showed widely distributed tracer uptake. It was shown that [^18^F]**32** crossed the BBB and was rapidly metabolized to [^18^F]fluoroacetate in both the brain and the peripheral tissues.^139^ However, injection of authentic [^18^F]fluoroacetate showed that the radiolabeled HDAC release product did not remain metabolically trapped in specific brain regions, meaning the background signal was very high. Blocking studies with known HDAC inhibitors did demonstrate that some of the tracer accumulation in the brain reflected HDAC activity. Through careful kinetic modeling and accounting for the nonspecific background signal generated by leakage of [^18^F]fluoroacetate away from sites of enzyme action, it was subsequently shown that [^18^F]**32** produced regions of specific uptake in the brains of monkeys. Postmortem histochemical analysis of brain tissue samples revealed that regions of high tracer uptake correlated with HDAC 2a expression, showing that with careful kinetic modeling [^18^F]**32** could be used to monitor HDAC expression in the brain.^140^ To date, no studies with [^18^F]**32** in humans have been reported. The ^18^F-labeled difluoro- and trifluoroacetate ([^18^F]**33** and [^18^F]**34**, respectively) analogues were also prepared with the goal of increasing signal sensitivity. Preparation of fluorinated derivatives would lead to lower pK_a_ values of the carboxylic acid product after HDAC enzymatic cleavage (Figure 22) and thus greater ionization at physiological pH and therefore reduced membrane permeability and increased accumulation of the radioactive acetate at the site of HDAC activity.^141^ However, since no in cellulo experiments demonstrating reduced cell permeability or in vivo experiments measuring time dependence were reported, it remains unclear whether increasing fluorination would lead to an improvement in image quality through improved metabolic trapping. Interestingly, the ^11^C-labeled analogue (**35**) was also prepared but was unable to cross the BBB.^142^ The reason why the relatively conservative change from a C-[^18^F]F in [^18^F]**32** to a C–H bond in [^11^C]**35** leads to a change in BBB permeability is not apparent.

**Figure 24.**
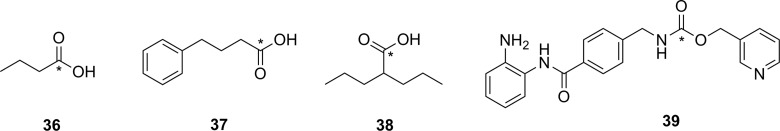
Potential imaging agents for HDAC based on clinical or preclinical drugs. *^11^C. HDAC indicates histone deacetylase.

Approaches employing reversible inhibitors of HDAC have shown varied success. All of the potential imaging agents based on reversible inhibition of HDAC have employed a functional group (hydroxamate or carboxylic acid) designed to coordinate to the active-site zinc ion found in class 1 and 2 HDACs. Known HDAC inhibitors have served as the starting scaffold for most radiolabeled HDAC inhibitors reported to date. For example, ^11^C-labeling of the pharmaceuticals butyric acid ([^11^C]**36**, Figure 24), 4-phenylbutyric acid ([^11^C]**37**), and valproic acid ([^11^C]**38**) followed by in vivo studies in baboons showed that despite their clinical use, all 3 had extremely poor penetration across the BBB with tracer accumulation predominantly in peripheral organs. This poor uptake in the brain was suggested as the possible reason that high doses of these drugs are needed to achieve neurological effects.^143^ Similarly, poor BBB penetrance was shown for an ^11^C-labeled analogue of MS-275 (entinostat, [^11^C]**39**), a compound in clinical trials for treatment of Hodgkin lymphoma, advanced breast cancer, and metastatic lung cancer. Labeling of **36** with ^11^C using [^11^C]CO_2_ fixation furnished the labeled compound, which was used to image HDAC in both rats and baboons using PET.^144^ In each case, ^11^C-radiolabeled versions of the clinical (**36**-**38**) or preclinical (**39**) drugs given at microdoses did not effectively cross the BBB, meaning they were not suitable for measuring HDAC expression in the brain. Interestingly, although [^11^C]**39** was not a suitable imaging agent, many derivatives of **39** have been prepared in an image-guided structure-activity relationship study to develop a tracer that could readily cross the BBB for imaging HDAC expression in the brain. This scaffold was chosen because derivatives could be prepared rapidly, in a modular fashion, and in reasonable yields allowing for rapid screening of candidate tracers. Radiolabeling was accomplished using [^11^C]CH_3_I, [^11^C]CH_3_OTf or [^11^C]CO_2_ as appropriate. The benefit of using ^11^C is its short half-life permitting multiple scans with different candidate tracers to be performed on the same animal in the same day, greatly increasing throughput, reducing cost, and reducing the number of test animals required. IC_50_ values were measured for nonradioactive analogues of each derivative in vitro and were in the range of approximately 1 to 100 nM, and PET imaging allowed a direct assessment of which compounds penetrated the BBB and to what extent. It was reported that in some cases, the measured IC_50_ values depended on the incubation period.^145^ This suggests that a more complete in vitro kinetic analysis of inhibitors with purified enzyme and measured *K_i_* values could lead to a better understanding of behavior in vivo, especially since the binding of benzamide inhibitors to HDAC has been shown to have slow-onset kinetics in some cases.^146,147^ The conclusion from preparation of derivatives of **39** showed that the overall polar surface area of the tracer needed to be <65 Å^2^ to easily cross the BBB. Many of the inhibitors used clinically as drugs do not meet this criteria, which likely explains their poor CNS distribution. Although regions of specific uptake of the PET signal in baboon brain were observed for many derivatives, the incomplete understanding of HDAC distribution within the brain, coupled with the poorly understood inhibitor kinetics, makes a complete interpretation of the imaging results difficult.^145^

**Figure 25.**
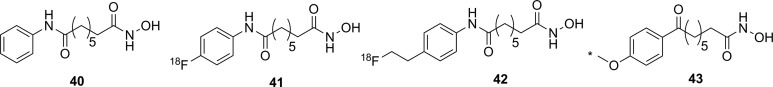
Suberanilohydroxamic acid (**40**) and radiolabeled derivatives (**41**-**43**) as potential imaging agents for HDACs. *^11^C. HDACs indicates histone deacetylases.

Derivatives of another compound in clinical use, suberanilohydroxamic acid (SAHA, vorinostat, **40** Figure 25) were also investigated as potential HDAC imaging agents in the brain. An ^18^F-labeled derivative ([^18^F]**41**) was prepared in a 4-step radiosynthesis, since attempts to directly introduce the [^18^F]fluorine onto the aromatic ring in a 1-step radiosynthesis were unsuccessful. Stability studies of [^18^F]**41** in rat plasma showed rapid metabolism and excretion, and PET imaging also showed significant uptake in kidneys and intestines. Although brain uptake was minimal, a xenograft ovarian cancer tumor in a mouse model did show some accumulation. This tumor accumulation could be blocked using unlabeled **40** (SAHA), suggesting that tracer [^18^F]**41** could be used as a diagnostic tool for imaging HDAC activity in peripheral tumors.^148^ [^18^F]**42**, a derivative with a [^18^F]fluoroethyl prosthetic group, was also prepared and evaluated both in vitro and in vivo. However, imaging studies and ex vivo biodistribution studies indicated significant uptake in the bones, intestines, kidneys, and liver. These results suggested metabolism and defluorination were both occurring to a significant extent, rendering [^18^F]**42** unsuitable for use as a tracer for HDAC activity.^149^ Finally, the O-[^11^C]methyl derivative **43,** which also lacks the internal amide between the flexible alkyl chain and the aromatic ring, was prepared and evaluated as a potential imaging agent for HDAC expression in the brain. However, [^11^C]**43** showed only nonspecific binding and no specific accumulation in baboon brain or peripheral organs that are known to express HDACs, meaning it was also unsuitable as an imaging agent.^142^

**Figure 26.**
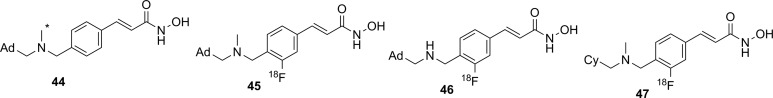
Martinostat (**44**) and derivatives for imaging of HDACs. *^11^C. Ad indicates adamantly; Cy, cyclohexyl; HDACs, histone deacetylases.

Hooker and coworkers have made the greatest progress toward an imaging agent of HDAC expression suitable for clinical use with Martinostat ([^11^C]**44**, Figure 26)^150^ and its derivatives (**45-47**).^151^ First reported in 2014, the development of ^11^C-labeled **44** is an excellent example of a carefully designed and executed process for creating a rationally designed PET imaging agent for an enzyme target in the CNS.^150^ [^11^C]**44** was based on a common HDAC inhibitor design: a zinc-binding group (hydroxamic acid) connected by a linker (cinnamic acid core) to a lipophilic cap group (adamantyl group). An adamantyl cap group was chosen because that group was previously shown to increase overall hydrophobicity and CNS permeability in a series of adamantyl-containing hydroxamic acid compounds targeting HDACs.^152^ IC_50_ values were measured for the nonradiolabeled analogue **44** as an inhibitor of HDACs in vitro, and it was observed to be a potent inhibitor (low nM) of classes 1 and 2b enzymes under the conditions tested. Further screening against 84 other zinc-containing enzymes and proteins showed no other significant binding, strongly suggesting **44** is very selective for classes 1 and 2b HDACs in vivo. Injection of [^11^C]**44** into rats followed by PET scanning demonstrated good uptake in the brain, which could be blocked in a dose-dependent manner by injection with cold **44**, SAHA (**40**), or another known inhibitor (CN54) specific to HDAC. In addition, [^11^C]**44** was shown to be metabolically stable in both rat and baboon plasma. Kinetic modeling suggested that between 50% and 80% of the signal arose from specific binding to HDAC,^150^ and a complete kinetic model for tracer distribution and uptake was subsequently developed.^153^ [^11^C]**44** was used to validate target engagement with brain HDACs using a number of known inhibitors that are potential therapeutics and explore potential mood-altering effects from HDAC inhibition.^154^ Tests in healthy human volunteers with [^11^C]**44** showed specific uptake in brain regions of interest, and no adverse effects were reported.^155^ Positron emission tomography scanning with [^11^C]**44** has enormous potential as a powerful tool for imaging of epigenetic changes associated with a number of disease processes. One drawback the authors noted in the use of **44** for imaging HDAC activity is the low radiochemical yield: It has been reported as between 3% and 5% nondecay corrected^150^ or as low as 1% to 2% on radiochemical scales suitable to prepare sufficient radiotracer for imaging in humans.^155^ Although sufficient levels can be produced to obtain a useful PET image of HDAC distribution, improvements in radiochemical yield are needed to reduce safety issues around handling large amounts of the starting material [^11^C]CO_2_. In part to address this need, the Hooker group also prepared 3 ^18^F-labeled derivatives (**45**-**47**). Compound [^18^F]**45** was a structural analogue of **44** with a relatively conservative aryl C–H to C–[^18^F]F modification, whereas the more hydrophilic derivative [^18^F]**46** lacked the *N*-methyl group and [^18^F]**47** had replaced the adamantyl group with the less lipophilic cyclohexyl moiety. The initial preparations of ^18^F-labeled **45** to **47** had radiochemical yields of <1% for [^18^F]**45** and [^18^F]**46** and 7% for [^18^F]**47**, all of which showed reduced uptake in baboon brain compared to [^18^F]**44**. Further studies such as biodistribution and metabolism studies will require an improved radiosynthesis.^151^

**Figure 27.**
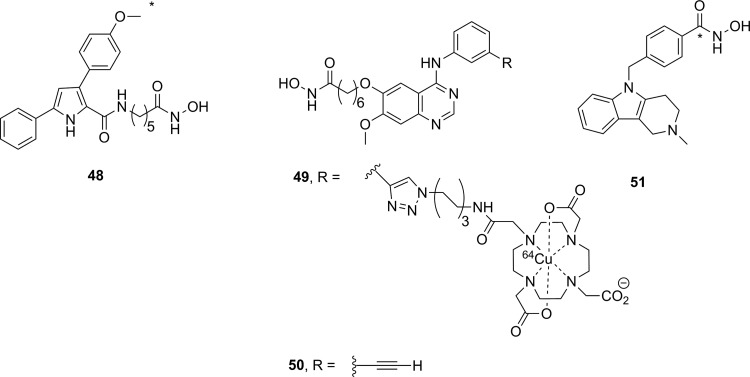
Other HDAC inhibitors and potential imaging agents. *^11^C. HDAC indicates histone deacetylase.

There have been three other radiolabeled inhibitors of HDAC that have been investigated as potential imaging agents. Compound [^11^C]**48** ([^11^C]Kendine 91, Figure 27) is an ^11^C-labeled version of a new class of HDAC inhibitor. The tracer was prepared to investigate the properties of **48** in vivo, as initial biological data for nonradioactive **48** as an anticancer therapeutic were promising. The authors reported that attempts to radiolabel the desmethyl precursor gave low radiochemical yields and multiple side products, attributed to side reactions involving the free hydroxamic acid functionality. No in cellulo or in vivo data were reported.^156^ The only example of an attempt to image HDAC activity using an inhibitor incorporating a metal-chelating agent was with **49**, a ^64^Cu-containing derivative of CUDC-101 (**50**), which is currently in phase 1 clinical trials for head and neck cancers. [^64^Cu]**49** was prepared by using a Cu-catalyzed [3+2] Huisgen cycloaddition to append the metal-chelating DOTA onto the alkyne in **50**. The large ^64^Cu-binding prosthetic group and linker were presumably solvent exposed, as [^64^Cu]**49** could still inhibit HDAC (IC_50_ = 0.09 μM), and cellular uptake could be blocked by addition of **50**. Injection into a xenograft mouse tumor model showed high uptake into the liver, kidneys, and the tumor. This tracer is currently undergoing further structural optimization to increase tumor uptake.^157^ Imaging agents of this class will be limited to imaging of HDACs in the peripheral tissues, since their large size and charge (−1 charge for Cu(DOTA), as shown in Figure 27) precludes crossing of the BBB. Finally, an initial attempt at preparing ^11^C-labeled tubastatin A ([^11^C]**51**), which is a specific inhibitor of HDAC 6, provided [^11^C]**51** in <10% radiochemical yield. No biological data for this tracer have yet been reported.^158^

### Fatty Acid Amide Hydrolase

**Figure 28.**
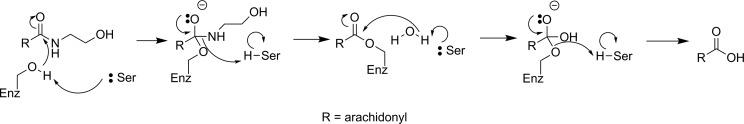
Mechanism for hydrolysis of anandamide by FAAH.^159^ FAAH indicates fatty acid amide hydrolase.

Along with MAGL (discussed above), FAAH is the other hydrolase enzyme known to play a major role in the endocannabinoid neurotransmitter system. The most important natural substrate for FAAH is thought to be anandamide (*N*-arachidonoylethanolamine), which is a retrograde lipid neurotransmitter^51^ and whose hydrolysis is depicted in Figure 28. Note that although FAAH is a serine hydrolase, it uses an unusual Ser-Ser-Lys catalytic triad.^159^ Inhibitors of FAAH are being investigated as tools to better understand the role of FAAH in disease or as potential therapeutics for conditions such as addiction, anxiety, schizophrenia, depression, and neurodegeneration.^160,161^ From a nuclear imaging perspective, FAAH is an interesting target since it is an intracellular target with a very hydrophobic substrate requiring a specific transport protein.^162^ Small molecule probes attempting to image FAAH activity and distribution have been developed using all 3 types of probe: substrates, reversible inhibitors, and irreversible inhibitors.

**Figure 29.**
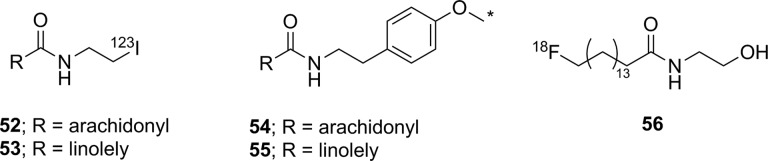
Potential substrates for SPECT (**52** and **53**) or PET (**54**, **55** and **56**) imaging of FAAH activity. *^11^C. PET indicates positron emission tomography; FAAH, fatty acid amide hydrolase.

The first attempt to image FAAH used ^123^I-labeled arachidonic and linoleic acid amide analogues (**52** and **53**, Figure 29) designed to image FAAH activity through metabolic trapping of the protonated iodoethylamine product. Both [^123^I]**52** and [^123^I]**53** were indirectly tested as substrates for FAAH using a competitive assay in which apparent IC_50_ values were measured in the presence of an authentic (tritiated) substrate.^163^ Unfortunately, such an assay does not distinguish whether a compound serves as a substrate or a competitive inhibitor for the enzyme and provides no detailed information on the kinetic efficiency of the putative substrate. Both tracers were injected into mice for biodistribution studies, and plasma stability studies clearly showed that deiodination was occurring rapidly, making both compounds unsuitable for imaging.^163^ The same authors also reported their attempts to develop a PET imaging agent using the same general approach that would not suffer from problems of deiodination with a modified scaffold (**54** and **55**). Following radiosynthesis with [^11^C]CH_3_I, ex vivo analysis and blocking studies in mice treated with [^11^C]**54** or [^11^C]**55** showed that both were processed in the brain by FAAH. However, brain uptake was low and tracer retention in both the blood and peripheral tissues was relatively high,^164^ meaning further optimization would be needed to generate a lead compound for imaging using this approach. Another attempt at creating an FAAH substrate to image activity based on a metabolic trapping approach was with radiotracer [^18^F]**56**. The probe was designed so that following hydrolysis by FAAH, the ^18^F-labeled fatty acid product would be incorporated into the cellular lipid pool and remain trapped in the cell membrane. Following radiosynthesis of [^18^F]**56**, the probe was injected into mice and ex vivo biodistribution analysis showed widespread and relatively uniform distribution in both the peripheral tissues and the brain. Pretreatment with a known FAAH inhibitor did not alter tracer biodistribution,^165^ demonstrating this approach appears unsuitable for imaging FAAH. Generally, the development of a substrate-based approach for imaging FAAH activity in the brain faces an unavoidable dilemma: The need to have the lipophilicity of the probe in a narrow range (log *P* between 2.0 and 3.5) to allow efficient crossing of the BBB without binding too tightly to plasma components^35,36^ balanced against the need for a large lipid tail to ensure adequate recognition of the substrate in the active sites of the enzyme and the binding site of the transport protein. As well, the difficulty in assaying artificial substrates to easily rank tracer candidates makes structural optimization another considerable challenge.

**Figure 30.**
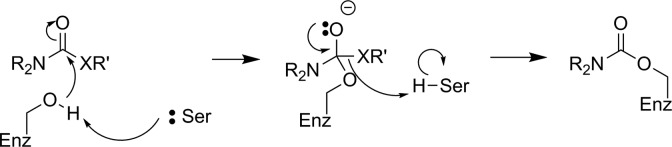
Reaction of FAAH with carbamate-based (X = O) or urea-based (X = NH) irreversible inhibitors to form a stable enzyme–inhibitor complex.^166^ FAAH indicates fatty acid amide hydrolase.

**Figure 31.**
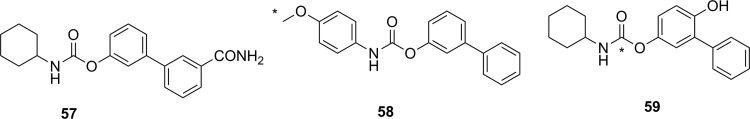
Known carbamate irreversible inhibitor for FAAH (**57**, URB597), and analogues (**58** and **59**) intended as PET imaging agents for FAAH. *^11^C. FAAH indicates fatty acid amide hydrolase; PET, positron emission tomography.

Most of the irreversible inhibitors for PET imaging of FAAH contain a carbamate or urea group that acylates the nucleophilic serine residue in the active site (Figure 30).^166^ The first attempt to image FAAH using an irreversible inhibitor was inspired by the known carbamate-containing irreversible inhibitor URB597 (**57**, Figure 31).^167^ Tracer [^11^C]**58**, an analogue loosely based on URB597, was synthesized and labeled with ^11^C at a newly introduced phenol moiety. An apparent IC_50_ value of 436 nM was measured showing that unlabeled **58** inhibited FAAH activity, although ex vivo analysis showed no steady-state accumulation of ^11^C-labeled **58** in the brain which would be expected of an irreversible inhibitor. Significant peripheral metabolism and low brain uptake were also observed, and testing the mechanism of inhibition showed **58** was in fact a noncovalent (reversible) inhibitor of FAAH.^168^ Another tracer based on the *O*-arylcarbamate scaffold that has been prepared is [^11^C]**59** (also known as [^11^C]CURB),^169^ which is the ^11^C-labeled analogue of the known irreversible inhibitor URB694.^167^ URB694 was chosen instead of the better studied URB597, since previous work had demonstrated URB694 has better distribution in the brain.^170^ In this instance, the tracer was structurally identical to the original inhibitor, thus complete kinetic analysis was unnecessary since the mechanism of inhibition and kinetic measurements had already been demonstrated for the nonradioactive analogue.^166^ [^11^C]**59** was injected into rats, and ex vivo biodistribution revealed that brain uptake was high in regions known to express FAAH, with good brain to blood ratios. Pretreatment with unlabeled **59** decreased uptake in the brain in a dose-dependent manner, and the radioactive signal did not leak out of the brain, consistent with irreversible inhibition. Analysis of metabolites in the blood demonstrated that while some polar metabolites (presumably incapable of crossing the BBB) were generated, none could be detected in the brain itself.^169^ Based on these encouraging results, [^11^C]**59** was next evaluated in human volunteers to develop a complete kinetic model. In the case of FAAH imaging with [^11^C]**59**, the scans in healthy volunteers demonstrated that tracer binding was not limited by cerebral blood flow, and rate constants for all of the important processes including delivery and covalent inactivation could be derived from the data.^171^ Following this, whole-body radiation doses were calculated in healthy human volunteers injected with [^11^C]**59** and shown to be acceptably low.^172^ Further test–retest experiments in healthy volunteers validated the kinetic model, and blocking studies with known inhibitors proved the specificity of [^11^C]**59** for FAAH in humans.^173^ Based on these results, [^11^C]**59** was used to examine individuals with a C385A polymorphism for FAAH and who have a greater risk of anxiety or addiction, in whom it showed reduced uptake in the brain.^174^ [^11^C]**59** is now poised to become a new standard research tool for PET imaging of FAAH distribution in the brain for studying a variety of conditions or new potential drugs.

**Figure 32.**
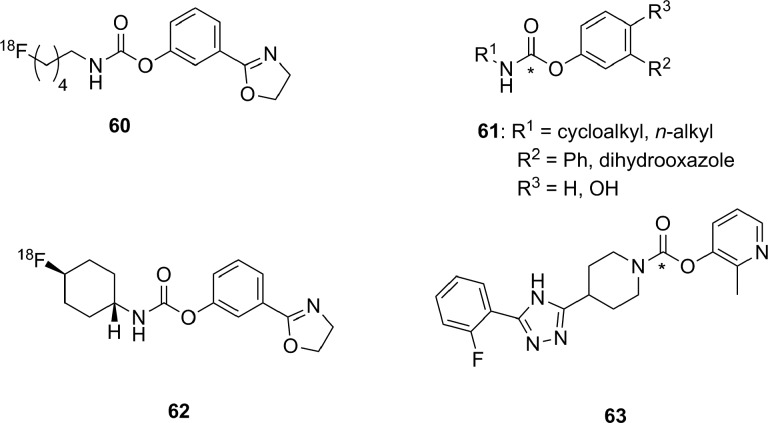
Other carbamate-based irreversible inhibitors for FAAH as potential PET imaging agents for FAAH. *^11^C. FAAH indicates fatty acid amide hydrolase; PET, positron emission tomography.

In addition to [^11^C]**59**, other carbamates have been prepared and tested as potential PET imaging agents for FAAH (**60**-**63**, Figure 32). ^18^F-labeled **60** was prepared and tested as an irreversible inhibitor of FAAH. The irreversibility of binding was shown by the fact that preincubation times affected the measured IC_50_ values and that injection of [^18^F]**60** into rats led to the radioactivity irreversibly binding to brain tissue. Binding to all tissues could be blocked by pretreatment with known FAAH inhibitors, demonstrating the specificity of binding. Interestingly, the compound was considerably less stable in rat plasma compared to human plasma, which was attributed to carboxylesterase activity against the dihydrooxazole moiety, which is found in rats but not in primates. Despite this increased sensitivity to degradation in rat plasma, most of the radioactivity detected in the rodent brain could be attributed to specific binding of [^18^F]**60** to FAAH and the uptake kinetics of the tracer into the brain were rapid.^175^ [^18^F]**60** was also examined in baboons, and metabolism studies supported the contention that rat plasma degraded **60** much more quickly than primates. Furthermore, these studies allowed full kinetic modeling of tracer biodistribution, which showed a 2-compartment model (similar to that used for [^11^C]**59**) best described the distribution of [^18^F]**60** in baboons.^176^ To further examine the potential benefit of incorporating a dihydrooxazole into a potential tracer, a series of 6 novel ^11^C-labeled carbamate derivatives were prepared (**61**) in which the alkyl groups appended to the nitrogen were varied, as were the *meta*-substituents on the phenol. Measurements of % FAAH inhibition at 4 concentrations showed all 6 were potent inhibitors of FAAH activity in vitro. Injection into rats followed by ex vivo biodistribution studies demonstrated that dihydrooxazole derivatives generally penetrated the brain better than the analogous phenyl inhibitors. The trade-off for this improved BBB permeability was the increased lability in rat plasma.^177^ Building on this work, an ^18^F-labeled dihydrooxazole carbamate probe ([^18^F]**62**) was prepared. Despite radiosynthetic challenges encountered when trying to perform an S_N_2 reaction at a secondary carbon to prepare an [^18^F]alkyl fluoride, [^18^F]**62** was successfully synthesized and studied in rats. Ex vivo biodistribution studies, PET imaging, and blocking experiments with **57** (URB597) demonstrated that [^18^F]**62** was highly specific for FAAH and gave very low background signal: Notably, it had rapid brain uptake and blood clearance and low nonspecific binding. Metabolism studies showed that the compound was degraded at a moderate rate in plasma, but the resulting metabolites were more hydrophilic than the parent tracer and thus did not interfere with brain imaging since they were unable to cross the BBB.^178^ These dihydrooxazole-containing carbamate inhibitors can incorporate either ^11^C or ^18^F into their design, and such compounds also appear to be promising potential FAAH tracers. The final carbamate-based inhibitor for FAAH that has been designed as a potential imaging agent is [^11^C]**63**, which was prepared in 20% radiochemical yield using [^11^C]COCl_2_.^179^ Although the authors suggest that this scaffold should be superior to **57** (URB597) because of a 2-fold better IC_50_ value, relying on IC_50_ values when comparing the effectiveness of irreversible inhibitors tested in different laboratories is of questionable value due to the time and experimental condition dependence of inhibition (as discussed in the Introduction section). Furthermore, the potency of an inhibitor is only one factor that must be balanced during the design of a successful imaging probe for enzymes found in the brain.^14,37,180^ Nonetheless, biodistribution studies in mice were promising and revealed that [^11^C]**63** did readily cross the BBB and tracer accumulated in brain regions of interest, which could be blocked by pretreatment with either unlabeled **63** or **57** (URB597). Further, the radiotracer did not wash out of the brain, suggesting irreversible inhibition of FAAH. Studies in monkeys showed similar results, although activity was conspicuously absent in the striatum, an area of the brain that is known to express FAAH^181^ and is clearly imaged with [^11^C]**59** ([^11^C]CURB). The reason for this difference is unclear. Activity in the monkey brain did not reach a plateau over time at high doses, suggesting moderate amounts of nonspecific binding. The authors concluded that a more hydrophilic analogue of [^11^C]**63** will need to be designed prior to studies in humans in order to minimize nonspecific binding.^179^

**Figure 33.**
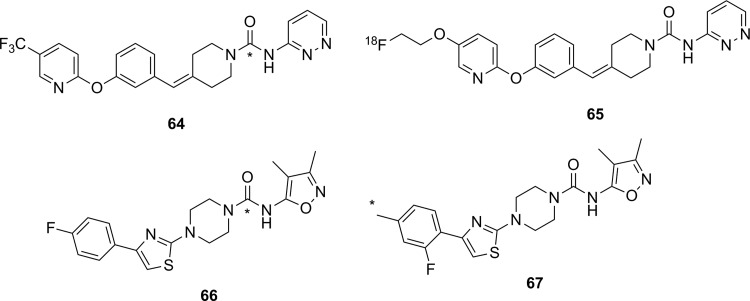
Urea-based irreversible inhibitors for FAAH as potential PET imaging agents for FAAH. * ^11^C. FAAH indicates fatty acid amide hydrolase; PET, positron emission tomography.

Four urea-based irreversible inhibitors of FAAH have been reported as potential imaging agents for FAAH. [^11^C]**64** (Figure 33) is the ^11^C-labeled analogue^182^ of PF-04457845, which is a compound currently being studied as a potential therapeutic for pain management.^183,184^ While synthesized in 5% radiochemical yield using direct fixation of [^11^C]CO_2_ in an automated synthesis module, sufficient material could be produced to study [^11^C]**64** in rats. The advantage of directly radiolabeling a pharmaceutical candidate is that the chemical structure is unchanged and therefore the animal biodistribution and toxicity data are already available for reference from the parent nonradioactive drug. Biodistribution studies were performed to evaluate brain uptake of radiotracer, including metabolite analysis and pretreatment experiments with either unlabeled **64** or **57** (URB597). In a direct comparison with [^11^C]**59** ([^11^C]CURB), it was determined that [^11^C]**64** showed greater specific binding to FAAH in the brain.^182^ Interestingly, [^18^F]fluoroethyl-modified derivative [^18^F]**65** was previously prepared as a PET imaging agent for FAAH based on PF-04457845. In the case of [^18^F]**65**, more significant radiosynthetic challenges had to be overcome. It was shown that [^18^F]**65** specifically reduced FAAH activity (and no other serine hydrolase) and in vivo studies in rats showed significant regions of uptake in the brain which could be directly blocked by preinjection with unlabeled **64** (PF-044578945).^185^ However, to date, no further progress on the development of a PET imaging agent for FAAH based on PF-044578945 has been reported. Finally, two urea-based irreversible inhibitors ([^11^C]**66** ^186^ and [^11^C]**67** ^187^) were separately prepared and studied in rats. In both cases, the major conclusion was that although specificity could be demonstrated and brain uptake was observed, the degree of nonspecific binding was significant, meaning further structure optimization to reduce hydrophobicity was needed.^186,187^

**Figure 34.**
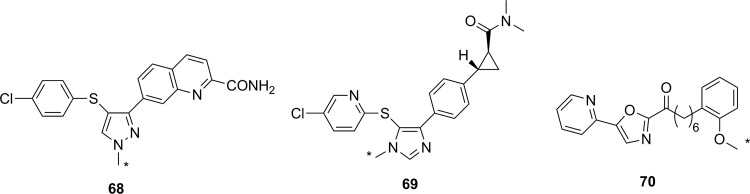
Noncovalent inhibitors used as PET imaging agents for FAAH. *^11^C. FAAH indicates fatty acid amide hydrolase; PET, positron emission tomography.

To date, three radiolabeled reversible inhibitors of FAAH have been reported as imaging agents of FAAH. The first two compounds, [^11^C]**68** and [^11^C]**69** (Figure 34), were first reported in 2012. [^11^C]**68** was studied as an imaging agent for FAAH distribution. However, the high lipophilicity (log *D* = 4.2) coupled with rapid metabolism of the compound led to undesired signals in the brain from lipophilic metabolites, making [^11^C]**68** unsuitable as an imaging tracer for FAAH. The second compound described, [^11^C]**69**, was developed after a structure optimization protocol attempting to reduce lipophilicity while still maintaining inhibitor potency. Unlabeled **69** was tested against a panel of 168 other possible protein targets (including other enzymes, ion channels, and receptors) and shown, on the basis of IC_50_ measurements, to be >1000-fold selective for FAAH in vitro. Injection of [^11^C]**69** into monkeys showed good metabolic stability and specific uptake in regions of the brain known to be rich in FAAH. This brain uptake could be blocked using unlabeled **69**, thereby demonstrating specificity.^188^ While the authors expressed the intent to begin PET imaging studies in humans using [^11^C]**69**, no subsequent reports have yet appeared in the peer-reviewed literature. The desire for a reversible inhibitor imaging agent for FAAH also motivated the development of [^11^C]**70** to serve as a tool for measuring enzyme availability as opposed to activity (described with irreversible inhibitors above). Based on modification of a known inhibitor of FAAH,^189^ [^11^C]**70** was produced and injected into mice. Ex vivo analysis in mice showed distribution in the expected organs based on known FAAH distribution and showed reversible binding in the brain. Positron emission tomography imaging in rats showed distribution similar to [^11^C]CURB ([^11^C]**59**) and [^11^C]**63**. While blocking studies with the known inhibitor **57** (URB597) showed [^11^C]**70** was interacting with FAAH, nonspecific binding in the brain was relatively high. Furthermore, the tracer was rapidly metabolized, albeit to hydrophilic metabolites unlikely to directly interfere with brain imaging. Experiments with knockout mice suggest that [^11^C]**70** is not cleared by efflux transporters (PgP or Bcrp), meaning this scaffold represents a promising lead for development of a more specific and more biologically stable noncovalent inhibitor for FAAH suitable for use as a PET imaging agent.^190^

## Conclusions and Perspectives

As described in this review, the most successful imaging agents have followed a development process somewhat akin to the following:

1. Identify an enzyme target linked to an important biological process (such as a disease) as a valuable imaging target.
2. Select a lead compound as a potential tracer. This is either through rational design of a new substrate/inhibitor molecule (such as Martinostat, [^11^C]**44**, for HDAC imaging) or starting with a previously reported lead compound described in the literature. Reports from pharmaceutical and biotechnology companies that list and describe drug candidates that either failed partway through the drug development process (such as SAR127303, **6**, for MAGL imaging) or are proceeding toward clinical use (such as PF-04457845, **64**, for FAAH imaging) have been particularly useful.
3. Measure the enzyme kinetics for the lead compound as a substrate, reversible inhibitor, or irreversible inhibitor in vitro. Specificity for the target enzyme is verified by demonstrating that the probe candidate is not efficiently recognized by similar or related enzymes through an in vitro screen using recombinant enzymes, cell lysate, or cell assays.
4. Optimize the lead compound structure for desirable properties such as in vitro efficiency and selectivity.
5. Perform cell-based studies for membrane permeability and intracellular target efficiency and selectivity. Such experiments can be greatly aided by employing genetic knockout techniques to create models that are either positive or negative for a given enzyme or small molecule inhibitors to reduce target activity in cellulo. For enzyme targets in the brain, performing an assay to model BBB permeability is also desirable.^191,192^
6. Prepare a radiolabeled analogue of the tracer candidate using convenient radiolabeling chemistry with sufficient radiochemical yields to provide enough tracer in high purity and specific activity for small animal studies.
7. Perform ex vivo biodistribution studies in a small animal model (typically a rodent) to determine modes and rates of clearance for the tracer, metabolic stability, and organ distribution and tissue or tumor uptake (in the case of oncology-based tracers).
8. Measure the specificity of the radiotracer for the target enzyme in vivo through co-injection of a nonradioactive analogue of the tracer with known binding to the target to saturate the target enzyme and block processing and/or retention of the tracer. Additional experiments to confirm specificity in vivo are helpful, such as correlating tracer uptake with direct measurements of enzyme expression levels (eg, Western blots) as well as using well-established co-injection of a known selective inhibitor of the target enzyme. Ex vivo tissue samples can also be evaluated by overlaying images from autoradiography and immunohistochemical staining to confirm co-localization of the target enzyme with the radioactive tracer.
9. The PET/SPECT imaging in a small animal model, both a disease model and a healthy control. Imaging results should be correlated with biodistribution studies, and additional blocking studies can also be performed with PET/SPECT imaging.
10. Especially for target enzymes found in the brain, metabolism and imaging studies with nonhuman primates.
11. Begin first in human studies, potentially advancing to clinical human trials.^2^

These development steps represent an ideal process that may not be feasible in all cases. Of course, careful planning of future steps can influence decision-making earlier in the process. For example, consideration of a planned radiosynthetic route can help guide the earlier, structure–activity optimization process. One common theme in the development of tracers that ultimately proved unsuccessful was a lack of experiments done in vitro and in cellulo to identify potential problems. Although tracer candidates that have shown great promise by thorough characterization in vitro are not guaranteed to have great in vivo imaging success, careful screening of early candidates can rule out poor candidates prior to expensive and often time-consuming radiolabeling and unnecessary animal studies (which has ethical considerations). In many of the unsuccessful attempts at radiotracer development for hydrolytic enzymes that failed in vivo, subsequent in vitro experiments revealed weaknesses and deficiencies that could have been identified prior to animal studies. For example, complete and appropriate enzyme kinetic evaluation of a tracer candidate should be performed prior to in vivo study to identifying unsuitable candidates, as well as in cellulo experiments to verify membrane permeability for intracellular targets or BBB permeability for targets in the brain. Additionally, in vitro experiments using purified enzymes to carefully delineate tracer specificity among isozymes would likely increase chances for success in vivo.

The majority of nuclear imaging agents for hydrolytic enzymes reported to date are reversible or irreversible inhibitors. This is particularly true for proteases (not discussed in detail herein). In contrast, there have been many fewer substrate-based imaging agents reported, and even fewer of those have proven successful. Unlike the development of an inhibitor, for which an in vitro enzyme assay is already established, most new substrates do not have *k_cat_* and *K_m_* values, nor experiments testing specificity over related enzymes, reported in the literature. Indeed, there are numerous difficulties and challenges in developing a new assay, such as using substrates without chromophores or fluorophores, solubility issues of some enzymes (ie, membrane bound or associated enzymes), and a lack of availability of purified enzymes. This is an unfortunate gap in knowledge: If a putative substrate-based imaging agent has suitable pharmacokinetic properties yet is unable to image the target enzyme, it can be difficult to determine whether the failure is a result of the substrate being kinetically incompetent at tracer levels or is processed by a similar (but off-target) enzyme. Another major challenge for substrate-based imaging agents has been the lack of a reliable metabolic or chemical immobilizing moiety that traps the radionuclide at the site of enzyme activity. Strategies relying on either hydrophobicity to lock the radioactive reporter in cell membranes or precipitation or the unmasking of an ionizable group to generate a cell membrane impermeable ion are clever, but this makes development of new tracers even more complicated and these methods have been met with variable success. Nonetheless, the development of a reporter group that can be readily attached to a substrate, does not interfere with recognition by the target enzyme, is easily radiolabeled, and reliably accumulates at the site of enzyme action would be a major advancement in developing substrate-based nuclear imaging agents for hydrolytic enzymes.

Just as the development of [^11^C]CO_2_ fixation technologies^56,193,194^ led to a surge in development of imaging agents for both HDAC and FAAH, other new radiosynthetic technologies are likely to drive the development of new tracers for imaging a wide array of hydrolytic enzymes. Exciting and convenient (“kit-like”) new approaches have been recently reported that could easily drive the future development of tracers for imaging hydrolytic enzymes, such as reactions with uncommon nuclei^195^ including [^18^F]trifluoroborates,^196-198^ [^18^F]silicon fluoride,^199,200^ [^18^F]sulfonyl fluoride-containing prosthetic groups,^201^ and chelation of Al[^18^F]F.^202^ The major drawback to many of these techniques as they currently exist is that they require attachment of prosthetic groups or chelators to facilitate radiolabeling, which are chemical moieties that add steric bulk and can change the polarity and biodistribution of the attached drug. Another promising area of future development are new reactions for rapidly forming aryl carbon–[^18^F]fluorine bonds.^203-205^

This is an exciting time in PET/SPECT radiotracer development for hydrolytic enzymes. There are now enough successful examples of tracer development to allow some reflection on promising strategies and an ideal process can be described. The development of new radiochemical methods will continue to drive innovation in tracer development. Finally, the rapid and successful development of imaging agents for both HDAC and FAAH shows the value of careful and thorough experimentation at each step of the development process. As well, involvement of a multidisciplinary development team that includes synthetic organic chemists, enzymologists, radiochemists, biologists, imaging scientists, and clinicians now appears to be critical for rapid success in taking an imaging agent from concept to human trials.
